# Advancements in 3D field-crop phenotyping using point clouds: a comparative review of sensor technology, target traits, and challenges under controlled and field conditions

**DOI:** 10.3389/fpls.2026.1731852

**Published:** 2026-02-06

**Authors:** Emmanuel Omia, Eunsung Park, Dennis Semyalo, Rahul Joshi, Byoung-Kwan Cho

**Affiliations:** 1 Department of Smart Agriculture Systems Machinery Engineering, Chungnam National University, Daejeon, Republic of Korea; 2 Department of Smart Agriculture Systems, Chungnam National University, Daejeon, Republic of Korea

**Keywords:** 3D crop phenotyping, laser triangulation, plant phenomics, precision agriculture, stereo vision, structured light, terrestrial laser

## Abstract

3D phenotyping refers to the quantitative characterization of a plant’s structural and morphological traits in three-dimensional space, allowing for a detailed analysis of plant architecture and growth patterns. In recent years, rapid advancements in non-destructive, high-throughput 3D imaging technologies have enabled the precise measurement of these traits. Initially focused on single-plant traits under controlled conditions, the field has now expanded towards robust applications in real-world field environments, enabling large-scale analyses of plant canopies and complex structures. This study focuses on the recent advancements in 3D crop phenotyping using point cloud technologies. It compares sensor technology and its application in controlled environments (Chamber-Crop Phenotyping, CCP) and field conditions (Field-Crop Phenotyping, FCP). Technologies such as Multiview stereo (MVS) reconstruction, LiDAR, and laser triangulation have enhanced plant phenomics by enabling high-throughput, non-destructive measurements of key traits such as canopy structure, leaf area, and stem diameter. This review highlights the strengths of the CCP, where environmental variables and flexibility are tightly controlled, facilitating precise trait measurement, and contrasts it with the challenges of the FCP, where unpredictable factors, such as occlusion, wind, light variability, and terrain complexity, complicate data acquisition. Various sensor platforms, including ground-based robotic systems and unmanned aerial vehicles (UAVs), have been discussed regarding their ability to overcome occlusion and limited sensor range in real-world conditions. The need to transition these technologies from laboratory environments to real-world agricultural applications is emphasized, highlighting their potential to improve crop management and plant breeding through accurate phenotypic trait extraction. Finally, current research gaps and future directions for integrating advanced sensor platforms and analytical techniques in both CCP and FCP settings are identified, emphasizing the need to enhance the scalability and robustness of 3D phenotyping for field applications.

## 1 Introduction

Geometry is fundamental to plant phenotyping, enabling the detailed analysis of plants’ morphological and structural traits in three-dimensional (3D) space (Bucksch et al., 2017). In recent decades, 3D point clouds have gained significant attention, particularly in the context of the Fourth Industrial Revolution, where they play a pivotal role in robotic vision perception and navigation (Lin and Juang, 2023). Today, almost every robotic system uses point clouds to interpret the complex geometries of its surroundings, facilitating safe and efficient navigation in human environments. This advancement has spurred the adoption of 3D point cloud technology in various fields, including agriculture (Jin et al., 2021). With the global population on the rise, there is an unprecedented demand for agricultural productivity and sustainable management of natural resources. Consequently, integrating 3D point cloud technology into traditional farming practices is becoming increasingly essential for boosting food production while minimizing the environmental impact of agriculture (Araus et al., 2022; Vandenberghe et al., 2018).

In agriculture, point cloud technology has been increasingly adopted for diverse applications, such as autonomous machinery navigation and plant phenotyping (Iqbal et al., 2020a). This study focuses on the latter and explores how point clouds transform modern plant phenomics. Plant phenomics aims to extract qualitative and quantitative traits to enhance and characterize plant phenotypes. Recent advancements in point cloud technology, combined with sophisticated data processing and analysis techniques, are poised to revolutionize plant phenotyping by improving the precision and ease of geometric-trait extraction. This high-resolution data are invaluable for breeders evaluating genotype performance in breeding plots and for farmers seeking precise crop management solutions. Researchers have actively conducted experimental studies to develop point-cloud-based methods, focusing on data collection platforms and analysis algorithms. However, most of these studies have been conducted in controlled growth environments, such as laboratories and greenhouses, rather than in open or semi-open field conditions (Langstroff et al., 2022). This is mainly because to the recommendation that initial research and development are recommended to be conducted in controlled settings to minimize variables and ensure reproducibility. However, real-world production environments present more complex challenges, requiring adaptations and modifications to these methods for successful field deployment (Araus and Cairns, 2014). Consequently, a comparative overview of advancements in 3D field crop phenotyping versus those conducted in controlled environments is essential.

To clarify, in this survey, phenotyping in controlled growth environments, such as laboratories and experimental greenhouses, is defined as Chamber-Crop Phenotyping (CCP), whereas phenotyping in large open fields or production greenhouses is referred to as Field-Crop Phenotyping (FCP). The key distinction between these categories lies in environmental variability and flexibility regarding the movement and/or rotation of individual plants or groups. CCP environments allow for greater environment control and flexibility, as plants are often grown in pots that can be easily repositioned. However, in FCP environments, crops are typically planted directly in the soil, making it cumbersome to manipulate individual plants, in addition to the variability in environmental variables. Nevertheless, sensor mobility remains comparable across both environments, except for the ability to deploy sensors at high altitudes (>10 m) in the FCP, which is typically not feasible in CCP settings. Planting patterns and density are also critical factors in categorizing phenotyping environments, as they influence the potential for occlusion and overlap. Crops planted under optimal production conditions, whether in open fields or greenhouses, are classified as FCP, as they prioritize yield and reflect real-world farming scenarios. Conversely, if plants are deliberately spaced to facilitate easy data collection at the expense of yield, they are categorized as CCP, even if grown in open-field breeding plots. Understanding these distinctions is crucial because planting density and pattern can significantly impact the range of measurable phenotypic traits and the design of analytical algorithms.

In CCP settings, 3D point cloud measuring techniques offer unparalleled precision and control over environmental variables. Researchers can easily manipulate a plant’s positioning and/or rotation, lighting, and other parameters to optimize data acquisition. As a result, techniques such as structured light scanning (Rosell-Polo et al., 2015), laser scanning (Jin et al., 2021), and photogrammetry (Zhang and Zhang, 2018) have flourished, enabling high-resolution reconstructions of plant morphology with exceptional accuracy. However, the confinement of experiments to controlled environments may inadvertently limit the generalizability of these results to real-field production scenarios (Langstroff et al., 2022; Polder and Hofstee, 2014). In contrast, FCP presents many challenges, including variability in environmental conditions, illumination, terrain, occlusions, and extensive plant populations. These factors introduce complexities not encountered in laboratory settings, necessitating the adaptation of 3D point cloud techniques to accommodate real-world conditions. Additionally, If the sensor is mounted to a mobile agricultural machine, it must withstand mechanical vibrations and shocks in addition to atmospheric distortions such as moisture, dust, varying temperatures, and bright sunlight (Ninomiya, 2022). While the potential benefits of field-based phenotyping are substantial, the practical implementation of 3D imaging technologies in such environments requires careful consideration of these challenges.

This survey provides a comprehensive comparative review of 3D phenotyping techniques under both CCP and FCP conditions and evaluates their suitability across varying environments. By synthesizing the existing literature, the strengths, limitations, and research gaps of 3D point cloud measurement techniques in both settings are highlighted, focusing on transitioning innovations from controlled environments to real-world agricultural applications. This emphasis on field-based research and development is intended to facilitate the integration of advanced phenotyping technologies into crop production. This review explores advancements in 3D sensing and measurement techniques, the carrier platforms used across environments, and key phenotypic traits for genotype evaluation and precision crop management, concluding with a discussion of the prospects for 3D crop phenotyping using point cloud technology. For details on the processing and analysis techniques, a recent review by Harandi et al. (2023) is recommended.

## 2 3D vision techniques used in phenotyping

Obtaining precise quality 3D measurements of plant organs and/or structures, such as leaves, stems, and canopies, also known as 3D high-throughput plant phenotyping (HTPP), relies solely on the point cloud quality used in the process. 3D point cloud measurement techniques have gained significant attention in plant and crop phenotyping (Araus et al., 2022). This growing interest stems from the potential of 3D point cloud technologies to provide detailed spatial information on plant structures, facilitating comprehensive analysis and characterization (Harandi et al., 2023; Jin et al., 2021). However, while considerable research has been devoted to achieving more measurable traits under laboratory-based setups, the translation of these techniques to actual field conditions remains relatively underexplored and is limited to a few traits (Langstroff et al., 2022). This section aims to bridge this gap by conducting a comparative literature study focused on 3D plant and/or crop phenotyping techniques, particularly clarifying the strengths and weaknesses of various measurement techniques under both CCP and FCP environments. This section excludes techniques for anatomical-level phenotyping aimed at retrieving 3D internal structures, such as Magnetic Resonance Imaging (MRI), Positron Emission Tomography (PET), and Computer Tomography (CT), and only concentrates on external morphology extraction techniques, such as laser scanners and photogrammetry.

To facilitate a systematic comparison across technologies and environments, this review adopts a multidimensional evaluation framework. Following the comparative approach outlined by Paulus (2019), each 3D sensing technology was evaluated based on six key criteria: (i) achievable accuracy and resolution, (ii) measurable phenotypic traits, (iii) data acquisition throughput, (iv) platform compatibility and constraints, (v) dominant error sources, and (vi) environmental robustness for CCP versus FCP deployment. This framework enables a structured comparison of technologies that differ fundamentally in their operational principles but serve similar phenotyping objectives. **Table 1** summarizes these characteristics across all the technologies discussed in this review, providing a consolidated reference for researchers and practitioners selecting appropriate sensing solutions for their specific phenotyping requirements. The following subsections detail each technology according to this framework, with explicit attention to the comparative performance under controlled and field conditions.

**Table 1:** Comprehensive comparison of 3D sensing technologies for plant phenotyping under CCP and FCP conditions.

Technology	Environment	Achievable accuracy	Key measurable traits	Throughput	Platform compatibility	Dominant error sources	Representative Studies
Laser Triangulation (LTS)	CCP	14 µm–45 µm resolution; R² >0.85 for leaf area	Petal thickness, leaf area, plant volume, ear volume, organ-level geometry	Low (single plant)	Articulated arm, turntable, fixed mount	Chlorophyll absorption at 660 nm, leaf translucency, edge effects	Lee et al. (2013); Paulus et al. (2013); Cai et al. (2020)
	FCP	cm-level; R² = 0.80–0.99 for canopy traits	Canopy height, biomass density, leaf area index	Medium (gantry-based)	Gantry systems, mobile platforms (range 0.8 m–2.4 m)	Limited range, occlusion, dust, platform vibration	Ehlert et al. (2008); Vadez et al. (2015); Virlet et al. (2016)
Multiview Stereo (MVS)	CCP	mm-level; R² = 0.87–0.99 for height/leaf area	Plant height, leaf area, stem diameter, 3D architecture	Medium (1–2 min/plant)	Multi-camera rig, turntable, robotic arm	Texture-less regions, processing time, calibration errors	Kumar et al. (2014); Rose et al. (2015); Wu et al. (2020)
	FCP	cm-level; R² = 0.78–0.99 for canopy traits	Canopy height, plot-level biomass, leaf angle, row structure	High (hectares/hour via UAV)	UAV, ground robot, handheld	Wind-induced motion, variable lighting, dense canopy occlusion	Klodt et al. (2015); Bao et al. (2016); Xiang et al. (2023)
Time-of-Flight (ToF)	CCP	cm-level; 10%–14% mean error for canopy traits	Plant height, stem diameter, canopy volume, dynamic growth	High (real-time)	Ground robot, UAV, tractor-mounted	Sunlight saturation, limited range, motion artifacts	Ruckelshausen et al. (2009); Young et al. (2019); Fan et al. (2022)
	FCP	cm-level; 10%–14% mean error for canopy traits	Plant height, stem diameter, canopy volume, dynamic growth	High (real-time)	Ground robot, UAV, tractor-mounted	Sunlight saturation, limited range, motion artifacts	Ruckelshausen et al. (2009); Young et al. (2019); Fan et al. (2022)
Terrestrial Laser Scanning (TLS)	CCP	sub-mm to mm; R² >0.90 for organ traits	Detailed plant architecture, leaf traits, organ segmentation	Low-Medium (static scanning)	Tripod, fixed post	Occlusion requiring multiple positions, cost	Wang et al. (2018); Panjvani et al. (2019); Patel et al. (2023)
	FCP	cm-level; RMSE 5 cm–6 cm for plant height	Canopy height, biomass, structural metrics, plot-level traits	High (mobile/backpack)	Tripod, vehicle-mounted, backpack, UAV	Ground-canopy separation, wind sensitivity, point density variation	Friedli et al. (2016); Deery et al. (2020); Pan et al. (2022)
Structured Light (SL)	CCP	mm-level; <13 mm error; R² >0.9 for leaf area	Leaf area, stress response, high-resolution surface geometry	Medium (single plant)	Fixed setup, controlled lighting required	Ambient light interference, calibration sensitivity	Nam et al. (2014); Nguyen et al. (2015, 2016a)
	FCP	cm-level; R² = 0.99 under low ambient light	Canopy structure (low-light conditions only)	Low (dawn/dusk/night only)	Mobile platform (restricted operation)	High ambient light severely degrades performance	Rosell-Polo et al. (2015)
Light Field (LF)	CCP	Limited depth range (10 cm–50 cm); qualitative	Stem and leaf morphology, post-capture refocusing	Low (large files, heavy processing)	Fixed mount, careful calibration required	Limited FOV, depth resolution, calibration complexity	Polder and Hofstee (2014)
	FCP	Limited; 4.33 average deviation error for height	Plant height (short-range only)	Very Low	Ground robot (experimental)	Short effective range, computational requirements, cost	Schima et al. (2016)

Accuracy values represent typical ranges reported in literature; actual performance varies with sensor model, plant species, and experimental conditions. R² values indicate correlation with manual/reference measurements. CCP, Chamber-Crop Phenotyping; FCP, Field-Crop Phenotyping. Throughput categories: Low (<10 plants/h), Medium (10–100 plants/h), High (>100 plants/hour or continuous field coverage).

### 2.1 Laser triangulation

**Figure 1:**
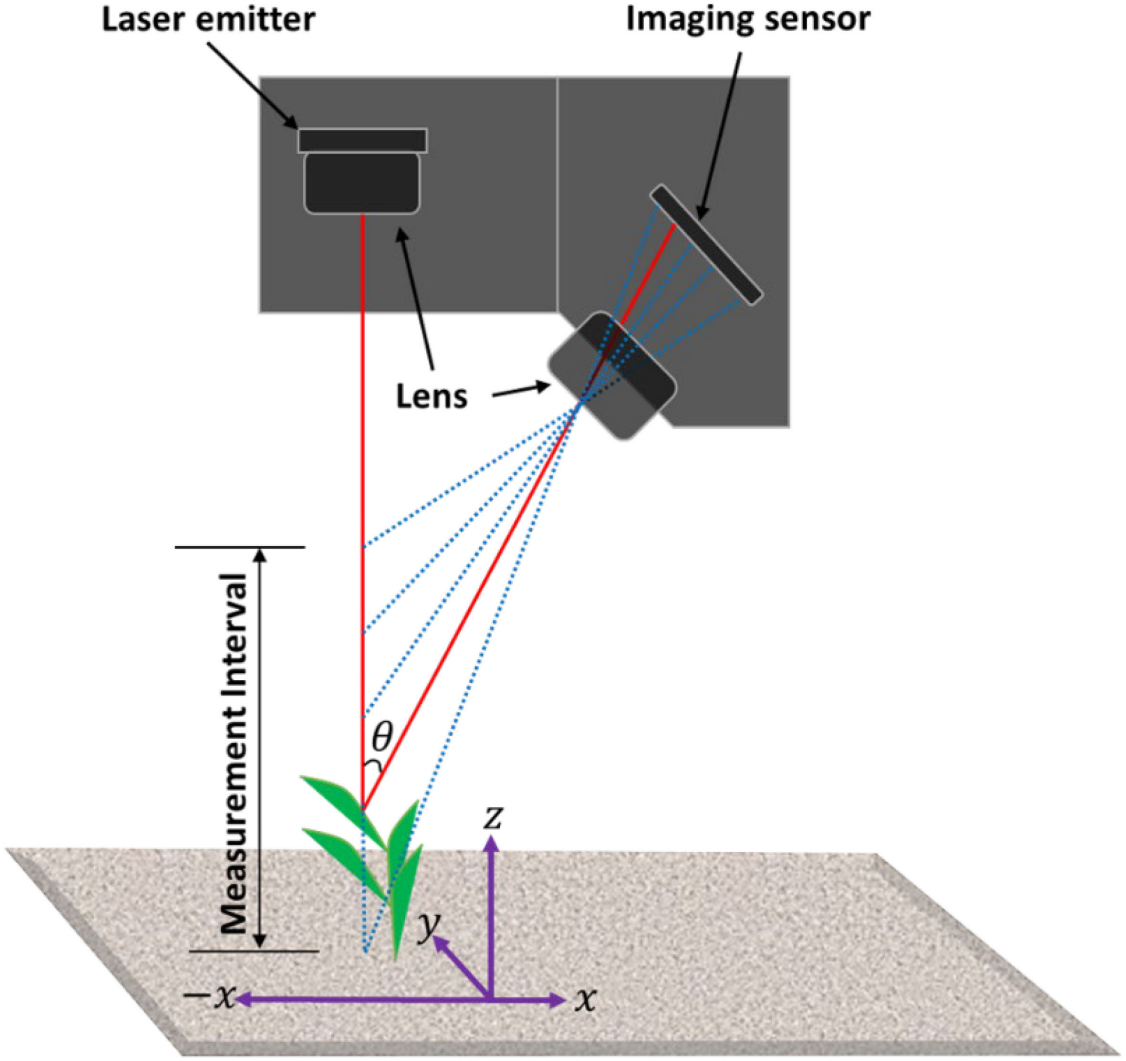
Laser triangulation measurement principle: A laser beam (red) is projected onto the target surface, and the reflection angle (θ) is determined with respect to the imaging sensor.

One of the most employed principles in low-cost 3D measurements is laser triangulation scanning (LTS) owing to its straightforwardness and robustness. The design of the scanner relies on basic trigonometry. Laser triangulation employs non-contact optical methods to capture accurate 3D dimensional information of an object or its surface. It operates based on trigonometric triangulation, projecting a laser beam onto the target surface at a predetermined angle with respect to an imaging sensor or camera. The core principle of LTS is the projection of a laser beam onto the target surface at a calculated angle relative to an imaging sensor (**Figure 1**). As the reflected laser light interacts with the surface, intricate details are captured and analyzed, enabling accurate spatial 3D reconstructions. This method is prized for its high precision (in µm) and low cost, making it an attractive option for phenotyping plants. Malhotra et al. (2011) and Schlarp et al. (2019) detailed the principles and algorithms underpinning laser triangulation, emphasizing its capability to achieve high-resolution 3D profiling with an accuracy range of 15 μm, as noted by Dupuis and Kuhlmann (2014). Its rapid data acquisition and non-destructive nature make laser triangulation ideal for high-throughput phenotyping platforms, facilitating continuous monitoring of plant development and stress responses.

For CCP applications, LTS has primarily been utilized to obtain precise 3D measurements of various plant traits, facilitating the analysis of individual plant growth, health, and other phenotypic characteristics. One of the key applications of laser triangulation in plant phenotyping is the accurate measurement of plant volumes and surface features. For instance, the 3D reconstruction of plants, such as potatoes, can be achieved through laser triangulation, allowing for precise volume measurements. This is particularly beneficial for phenotyping tasks, such as grading potatoes based on their size and weight, which are directly related to their volume (Cai et al., 2020). According to Cai et al. (2020), their process involved using a monocular camera and a line LTS laser to scan the surface of the potato and capture detailed coordinates that were used for 3D reconstruction and volume calculation.

Another important application is the measurement of leaf and petal thickness, which are critical indicators of plant health. Traditional contact-based methods often damage the delicate leaf and petal surfaces. In contrast, laser scanning provides a non-contact solution that can accurately measure these parameters in real time. For example, a device using dual laser triangulation was used to measure the thickness of Phalaenopsis petals with a resolution of 2 μm/pixel and a total measurement uncertainty of less than 16 μm (Lee et al., 2013). Their approach employed a cubic spline technique to fit the measured points on the petal surface, ensuring precise thickness calculation.

Moreover, laser triangulation is employed in high-throughput CCP to evaluate the geometric parameters of plants, such as the shapes and sizes of leaves and other organs. It allows the classification and parameterization of plant parts by analyzing 3D point clouds generated from laser scans. These point clouds can be processed to extract surface features and differentiate between various plant organs. The high automation and accuracy of this approach make it suitable for large-scale phenotyping studies, where quick and reliable data acquisition is essential (Paulus et al., 2013).

Although to a lesser extent, for FCP applications, several studies have highlighted the effectiveness of LTS in capturing detailed phenotypic data essential for understanding plant responses in their natural environments, mostly presented as integrated phenotyping platforms. One notable application is the LeasyScan platform (Vadez et al., 2015), which combines 3D LTS imaging with lysimetric measurements to assess canopy traits affecting water use, such as leaf area and transpiration rate. This platform continuously captures leaf area development and integrates gravimetric data, providing high-throughput and precise measurements that are critical for drought adaptation studies. Under field conditions, the LeasyScan platform achieved strong correlations between scanned and observed leaf area data (R² = 0.80–0.99 across various crops), with a measurement precision within 5% of the reference values (Vadez et al., 2015). Similarly, the Field Scanalyzer platform reported plant height estimation accuracy with an RMSE of 1.88 cm and R² = 0.97 when validated against manual measurements (Virlet et al., 2016). These results demonstrate the potential of LTS-based gantry systems to achieve centimeter-level accuracy in field phenotyping when the sensor-to-canopy distance is controlled.

Similar to LeasyScan, the Field Scanalyzer (Virlet et al., 2016) is an automated robotic field phenotyping platform. It employs a comprehensive sensor array, including dual 3D LTS scanners, to monitor crop performance at high temporal and spatial resolutions. This platform facilitates detailed measurements of canopy development and growth stages throughout the crop life cycle. By integrating multiple sensors, the Field Scanalyzer provides a robust dataset that supports the identification of key growth stages and specific growth measurements, contributing to more precise crop monitoring and breeding efforts. Ehlert et al. (2008) further demonstrated that vehicle-mounted laser scanners could estimate crop biomass density in field trials with R² values ranging from 0.93 to 0.99, though accuracy decreased at plant densities exceeding 200 plants/m² due to mutual shading and occlusion effects.

Although laser triangulation offers significant advantages for 3D phenotyping of plants, several studies have highlighted its limitations. These limitations can be inherent in the hardware capability or originate from the interactions between the laser system, plant characteristics, and specific measurement environment challenges. The most well-known hardware inherent limitation of LTS is short range (usually within a few ten to hundred centimeters), such as LASE ODS 1600 HT 2 (Danish company, LASE^®^) with a measuring range of 0.80 m–2.4 m (Ehlert et al., 2008). Paulus et al. (2014) investigated the influence of species, leaf chlorophyll content, and sensor settings on the accuracy of a 660 nm active laser triangulation scanning device. They found that the accuracy of the surface images varied significantly with leaf chlorophyll concentration and sensor exposure time. For example, the leaves of *Ficus benjamina* with low chlorophyll concentrations and long sensor exposure times yielded inaccurate surface images. Conversely, the rough, waxy surface of leeks (*Allium porrum*) can be accurately imaged using very low exposure times. However, longer exposure times result in penetration and multiple refractions, preventing accurate surface imaging. These findings suggest that plant properties and sensor settings must be carefully considered to achieve high accuracy in laser imaging for tasks such as monitoring plant growth and assessing responses to water stress.

Dupuis et al. (2015) examined the impact of different leaf surface tissues on the accuracy of 3D laser triangulation measurement. They compared two triangulation-based 3D laser scanners with different wavelengths (658 nm red and 405 nm blue) and found that the intensity of reflection from backscattered laser rays provided valuable insights into both the geometric accuracy and physiological conditions of plants. The study revealed that red lasers showed high interpretability in terms of tissue composition, whereas blue lasers provided higher geometric accuracy. However, the interaction with leaf tissues and the resulting absorption of the laser can affect the measurement accuracy. The ability to identify plant diseases, such as powdery mildew, and analyze tissue composition and leaf senescence stages using intensity data was demonstrated. However, these interactions highlight the need for further refinement of laser triangulation to achieve precise plant phenotyping.

Klapa and Mitka (2017) discussed the edge effect, a measurement error arising from the reflection of the laser beam on adjacent walls or its diffraction at the edges. This effect leads to incorrect positioning of points in space due to the averaging of measurements from multiple areas. This study presents case studies showing the discrepancy between the corner points in the models and the actual curved surface of the point clouds. This edge effect can significantly impact the quality and accuracy of measurements, emphasizing the need for improved methods to mitigate such errors in 2D and 3D laser scanning of plant structures.

In summary, laser triangulation demonstrated markedly different performance characteristics in CCP and FCP environments. Under controlled conditions, LTS achieves exceptional precision (14 µm–45 µm resolution; Dupuis and Kuhlmann, 2014) and strong correlations with manual measurements (R² = 0.85–0.97 for morphological parameters; Paulus et al., 2014), making it well-suited for organ-level phenotyping of individual plants, including leaf area, petal thickness, and volumetric traits. However, these advantages are contingent upon the careful optimization of sensor exposure settings relative to leaf optical properties, particularly chlorophyll concentration and surface characteristics. In FCP settings, the utility of this technology is constrained by its limited operational range (typically 0.8 m–2.4 m; Ehlert et al., 2008), susceptibility to environmental interference (dust, vibration), and challenges with canopy occlusion. Field applications have therefore focused predominantly on gantry-mounted systems (e.g., LeasyScan, Field Scanalyzer), where controlled sensor-to-plant distances can be maintained, achieving R² values of 0.80–0.99 for canopy-level traits, such as leaf area index and biomass density. The key trade-off between environments involves precision versus scalability: CCP enables micrometer-level accuracy on individual organs, whereas FCP deployments sacrifice fine-scale resolution for plot-level throughput under the constraint of fixed infrastructure requirements.

### 2.2 Multiview stereo reconstruction

**Figure 2:**
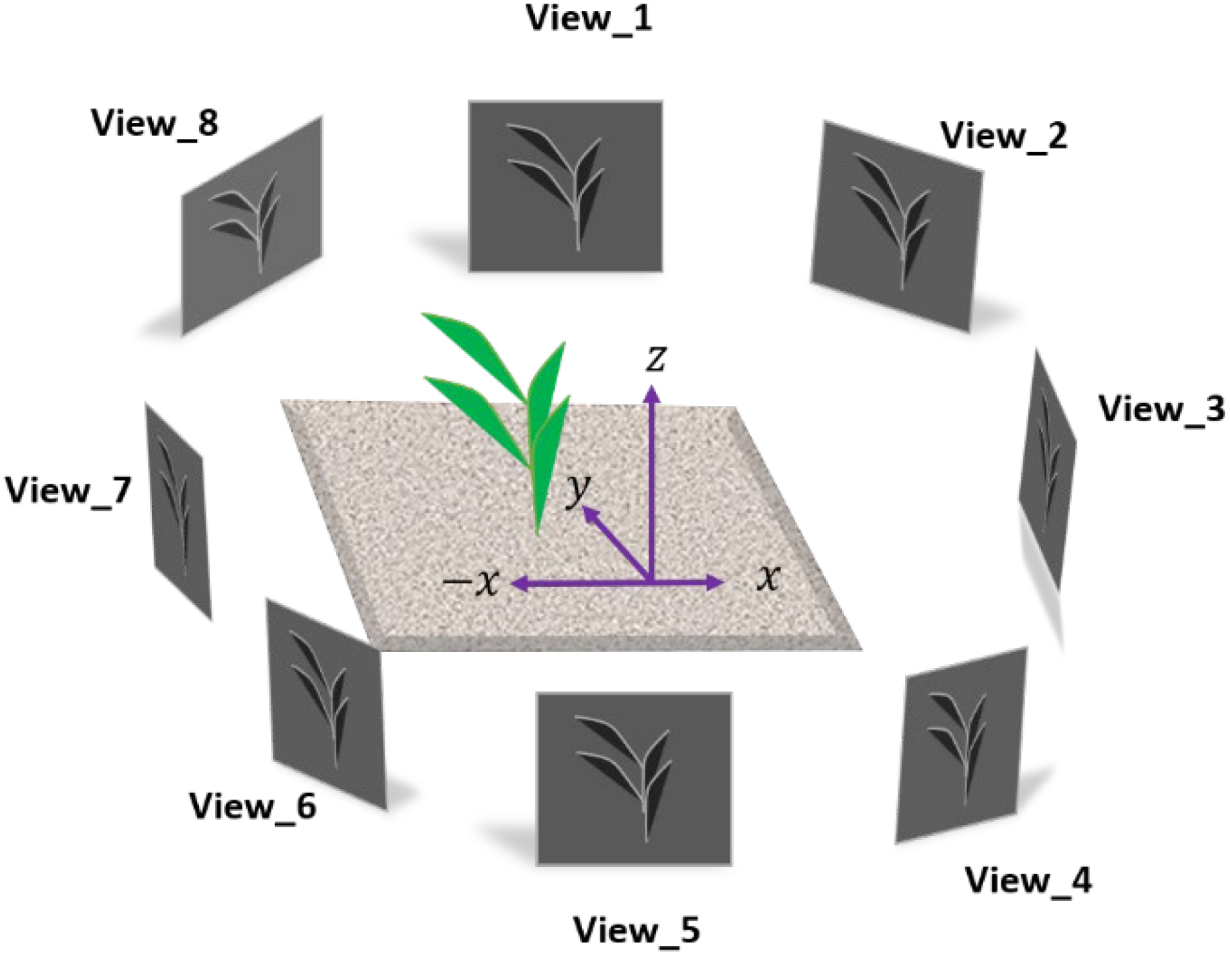
Multiview Stereo (MVS) Reconstruction: A series of images is captured from different viewpoints using multiple cameras around the target plant.

Multiview stereo (MVS) reconstruction is a well-established technology that has demonstrated significant potential for 3D modeling of plants since the mid-90s when the authors of Ivanov et al. (1995) first obtained an aerial 3D reconstructed model of maize canopy, enabling them to estimate leaf position and orientation and leaf area distribution. MVS takes advantage of multiple cameras positioned around the target plant to capture images from different viewpoints (**Figure 2**), which are then processed to reconstruct a 3D point cloud representation of the plant structure. This technique offers several advantages over traditional 3D scanning methods, including the ability to capture data rapidly and the use of relatively low-cost camera equipment (Nguyen et al., 2016a; Wu et al., 2020), making it relatively affordable and easily scalable with minimal overhead. It is evident in the literature that MVS has been widely used for phenotyping single plants under CCP compared to multiple plants in open-field conditions (FCP). Several aspects of MVS have been investigated in these studies to ascertain its suitability and competitiveness with other approaches.

The most attractive aspect of MVS for CCP and FCP applications is its cost. This is because MVS often takes advantage of readily available consumer color cameras, which are passive and thus require no extra lighting system, except in some studies that explore the use of structured light in combination with stereo cameras (Nguyen et al., 2016a). Lou et al. (2014) investigated the accuracy of multiview stereo 3D reconstruction for cost-effective, non-destructive plant phenotyping.

The experiment was conducted under CCP conditions, and each plant was scanned individually. Their main contribution was the development of a dense 3D reconstruction method that excelled in producing accurate 3D point clouds of various plants while retaining colors, textures, and shapes, compared to the earlier proposed methods by Furukawa and Ponce (2009) and Jancosek and Pajdla (2011). However, their approach still suffers from the effects of occlusions, texture-less regions, and blurred images, resulting in significant gaps or holes in the final 3D model of the plants. Additionally, the proposed method was relatively slow as the number of images to be processed increased. Similarly, Li et al. (2017); Rossi et al. (2020), and Wu et al. (2020) presents related narratives around the cost-effectiveness of the MVS phenotyping approach under CCP conditions. Collectively, these studies highlight the significant cost advantages of MVS phenotyping systems, while maintaining high accuracy and efficiency. The first study demonstrated a low-cost, portable stereo vision system that utilized high-definition webcams costing less than $70 and a laptop, and employed advanced algorithms to achieve robust and accurate 3D imaging under varying illumination conditions. Similarly, the MVS-Pheno platform automates image capture and 3D reconstruction of maize shoots in the field, combining affordability with high-throughput efficiency and strong correlations with manual measurements. Finally, the evaluation of a platform using low-cost sensors and cameras showed high accuracy in extracting morphological traits for multiple crops, reinforcing the potential of MVS systems for high-resolution plant phenotyping at minimal cost. These studies illustrate how MVS technology democratizes access to advanced phenotyping, enabling broader agricultural research and practice adoption by leveraging affordable hardware and sophisticated algorithms, without compromising data quality.

Although cost-effectiveness has dramatically inspired the adoption of MVS for 3D plant phenotyping, there are some associated drawbacks that are inherently to this technology. One such drawback is the compromise in processing speed because MVS requires secondary algorithms to convert image pairs into 3D point clouds. This introduces a processing overhead and can potentially require advanced computing capabilities to run the underlying reconstruction algorithms in cases where high-definition images are captured. Studies such as that of Kumar et al. (2014) have attempted to improve this drawback under CCP conditions by designing an easy-to-use camera calibration for single-axis motion coupled with a visual hull algorithm (Laurentini, 1995) for 3D reconstruction. Their approach achieved significant improvement in retrieving phenotypic quality 3D volumetric reconstruction with an acquisition time of less than a minute per potted plant compared to the previous study by Nguyen et al. (2016), which demonstrated an acquisition time of up to 2 min.

Second, the processing time required is the issue regarding the point cloud resolution/quality retrieved via MVS setups. Although MVS systems, particularly low-cost ones, can be limited in point cloud resolution due to factors such as camera quality, algorithmic challenges, baseline distance, environmental conditions, processing power, data coverage, and comparison to other high-resolution 3D imaging technologies such as LiDAR or structured light scanners, several studies have demonstrated that MVS can potentially attain high point resolution, especially when high-end hardware components coupled with complex algorithms are used (Klodt and Cremers, 2015; Rose et al., 2015). Moreover, Rose et al. (2015) demonstrated that the performance of the MVS system with high-end cameras competes favorably with the close-up triangulation line scanner Perceptron v5, which is superior in point accuracy and resolution of up to 14 µm. However, this exponentially increases the cost and computational complexity of MVS systems, which is undesirable for large-scale and real-time applications. Nevertheless, low-cost MVS systems remain valuable for many applications because of their affordability and flexibility. By understanding and mitigating these limitations in future studies, the resolution and accuracy of low-cost MVS-generated point clouds can be significantly improved, making them suitable for a broader range of phenotyping tasks, even under field conditions (FCP).

Some earlier studies have expressed reservations about the suitability of LiDAR for plant phenotyping. For instance, Lou et al. (2015) stated that “the 3D LASER/LIDAR scanner or the structured-light scanner (including Kinect sensor) do not work well on plants, especially on complex or even marginally occluded specimens or on small plants” (p. 555). While this assessment lacked experimental validation in their study, it likely reflected the genuine limitations of the LiDAR technology available at that time. Lin (2015), in a contemporaneous review, similarly acknowledged that “the currently-available LiDAR forms cannot effectively support the development of the next-generation techniques of plant phenotyping,” identifying the need for higher-density, full-waveform, and hyperspectral LiDAR variants then under development.

Several factors may have contributed to the challenges encountered with early LiDAR systems in plant phenotyping applications: (i) lower point densities in commercial terrestrial scanners circa 2010–2015, which were insufficient to resolve fine plant structures such as thin stems, small leaves, and complex branching patterns; (ii) limited algorithmic development for plant-specific point cloud processing, as early applications drew primarily from forestry and surveying domains where target geometries differ substantially from agricultural crops; (iii) the high cost of research-grade LiDAR equipment, which restricted access and limited systematic evaluation across diverse plant architectures; and (iv) specific experimental conditions, as performance varies considerably with plant species, growth stage, and scanning geometry.

Subsequent technological advancements have substantially improved the applicability of LiDAR to plant phenotyping. Higher-density scanning, multi-return signal processing, and dedicated algorithms for plant structure analysis have enabled successful applications across diverse crops, achieving centimeter-level accuracy for canopy traits and supporting large-scale field phenotyping (Jin et al., 2021; Zhu et al., 2021; Patel et al., 2023). The development of mobile and backpack-mounted LiDAR systems has further enhanced accessibility and throughput, addressing previous concerns regarding the practicality of this technology for routine phenotyping applications. Thus, while early skepticism reflected the real limitations of the technology at that time, the current state of LiDAR-based phenotyping demonstrates that these challenges have been substantially overcome through continued sensor development and algorithmic innovation.

Another aspect of MVS systems is the possibility of stereo spectral imaging, which makes it easy to render spectral information in 3D space, allowing for 3D plant health analyses and characterizations. This advantage is particularly interesting for field crop monitoring (FCP); however, the present studies have demonstrated it under CCP conditions only. For instance, Yoon and Thai (2010) presented an efficient approach to a stereo spectral imaging system for plant health characterization using a tunable stereo camera to switch between the NIR-band and Red-band alongside un-filtered raw stereo images. They used raw stereo images to match and reconstruct the 3D model of the plant, while the NIR and red-filtered stereo images were used for NDVI computation. Additionally, NIR stereo images were used for foreground object segmentation because background clutter was better suppressed by the NIR filter. Similarly, Santos et al. (2015) used spectral clustering to automatically segment plant leaves in point clouds. More details on spectral clustering can be found in Ng et al. (2001); Shi and Malik (2000), and Von Luxburg (2007).

Due to rapidly advancing hardware computational capabilities, MVS is increasingly integrated with robotic systems and advanced deep learning algorithms to improve plant phenotyping using camera-based approaches. The integration of MVS reconstruction with robotic platforms offers several key advantages for high-throughput plant phenotyping, including faster and more comprehensive data acquisition, reduced human labor, and the ability to capture 3D measurements of complex plant structures in their natural field environments (Dengyu et al., 2016; Gibbs et al., 2019). Similarly, the integration of deep learning techniques with MVS reconstruction has been shown to offer significant advantages for plant phenotyping applications. Deep-learning-based MVS approaches can automate critical tasks, such as feature extraction, cost volume regularization, and depth map inference, leading to more efficient and robust 3D plant model reconstruction compared to traditional MVS pipelines (Gao et al., 2024; Yang et al., 2024). These advancements facilitate high-throughput, precise, and nondestructive plant phenotyping measurements, which are key requirements for advancing modern agriculture and crop breeding efforts.

To this point, aspects and applications of MVS systems in plant phenotyping have been discussed, with most references focusing on studies conducted under CCP use cases. However, FCP often presents complex challenges, which makes it cumbersome to advance all possible CCP plant trait measurements to field conditions. The major limitation in the field is plant density, which results in heavy occlusion, making it challenging to image individual plants. For ground-based systems, researchers often extract target features on a row-plot basis instead of individual plants because of the severe occlusion caused by densely clustered leaves (Bao et al., 2016).

Additionally, plants in their natural habitat are fixed, so moving or rotating them is not an option to change their orientation. Therefore, coupled with the occlusion problem, the plant’s natural environment often presents a complex environment for FCP application. Moreover, the fields are usually large and consist of plant populations ranging from several hundred to millions of individual plants per acre, depending on the species. These complexities and the abundance of species in the field remain an underexplored challenge for phenotyping plants in their natural habitat. Several approaches have been adopted to minimize the effects of phenotyping plants in their natural habitat, including the use of mobile ground-based platforms, such as *Vinobot* in Bao et al. (2016) to capture organ-level phenotypic traits located at the middle and bottom of the plant canopy, fixed imaging towers, such as *vinoculer*, also described in Shafiekhani et al. (2017), and in some cases, UAVs (Di Gennaro and Matese, 2020) are employed to carry the imaging sensors to capture canopy-level information. Several other studies have also adopted robot-based platforms, such as the one designed by Bao et al. (2019a) to phenotype sorghum plant architecture, including plant height, stem diameter, leaf angle, leaf area, leaf number, and panicle size. Additional examples of robotics applications in field phenotyping can be found in the studies by Jay et al. (2014); Kim et al. (2021); Sodhi et al. (2017), and Xiang et al. (2023).

However, it is worth noting that occlusion is not always the major problem under field conditions, depending on the target crop species and growth stage. For instance, Klodt et al. (2015) used stereo reconstruction to estimate dense depth maps to distinguish grapevines in the foreground from other field plants in the background. Their objective was to correct the challenge of segmenting the foreground and background associated with RGB imaging of grapes in the vineyard.

Quantitative validation of MVS under field conditions revealed achievable accuracy levels that, while reduced compared to the CCP, remain suitable for many phenotyping applications. Klodt et al. (2015) reported RMSE values of approximately 3.0% for grapevine canopy volume estimation under field conditions. For cereal crops, UAV-based MVS achieves plant height estimation with R² = 0.91–0.98 and RMSE = 2.6 cm–9.0 cm, depending on flight altitude, camera specifications, and growth stage (Madec et al., 2017; Holman et al., 2016). Kim et al. (2021) demonstrated height estimation accuracy of R² = 0.78–0.84 for maize and sorghum under field conditions, with performance degrading at later growth stages due to increased canopy complexity. These studies indicate that MVS-based field phenotyping typically achieves centimeter-level accuracy for canopy-level traits, representing approximately one order of magnitude reduction in precision compared with controlled environment applications.

Furthermore, in the context of FCP, one of the challenges encountered is the impact of environmental factors, such as wind, which introduces a significant degree of uncertainty. Wind causes plants to move non-rigidly, resulting in dynamic and unpredictable motion. This complicates the application of traditional structure-from-motion (SfM) techniques, which generally rely on the assumption that the objects being analyzed are static or have minimal motion. Consequently, the inherent assumptions of these techniques are violated, making it difficult to accurately capture and analyze the crop structure in a natural field environment.

While most studies do not account for the effect of wind, some earlier studies, such as that of Biskup et al. (2007), attempted to mitigate this issue by stereomicroscopy of plants under outdoor conditions. This study emphasized the importance of synchronously triggering cameras for outdoor measurements, noting that plants are highly susceptible to wind and that successful stereo matching requires a rigid scene. Their field measurements with soybeans under various wind conditions demonstrated that reconstruction remained reliable in moderate wind and with a moving canopy; however, it failed in stormy conditions. This highlights the need for advanced techniques and considerations in FCP to handle the complexities introduced by environmental factors such as wind.

Expanding on this, a later study by Paturkar et al. (2019) investigated the effect of wind on the stereo reconstruction of plants under outdoor conditions. Their analysis revealed several adverse scenarios that present challenges and require further investigation. One such scenario involves acquiring images of plants in windy conditions, where plant movement leads to numerous feature-matching errors, resulting in poor 3D models. Specifically, the resulting models lacked essential details in the stem area and included only partially reconstructed leaves. A potential solution is to detect and filter out images with inconsistent matches caused by wind.

Additionally, this study explored the impact of changing light conditions, such as those caused by moving clouds. They found that drastic changes in illumination during image capture led to 3D models missing critical information about the plant surface and leaves, resulting in blank patches. To mitigate this issue, we proposed preprocessing and normalizing the acquired images to reduce the effects of illumination changes. These findings underscore the importance of addressing environmental factors such as wind and variable lighting in FCP. Overall, the development of MVS-based 3D reconstruction has been an essential advancement in plant phenotyping, providing a means to efficiently capture the detailed architectural traits of both laboratory-level and field-grown crops.

In summary, MVS reconstruction exhibited distinct advantages and limitations in phenotyping environments. In CCP settings, MVS systems achieve millimeter-level accuracy (R² = 0.87–0.99 for height, leaf area, and stem diameter; Rose et al., 2015; Wu et al., 2020) while maintaining cost-effectiveness through the use of consumer-grade cameras. The ability to control lighting, eliminate wind effects, and rotate plants for complete coverage enables high-fidelity 3D reconstructions that are suitable for detailed architectural analysis. Processing time (1 min–2 min per plant; Kumar et al., 2014) and computational demands represent the primary constraints, although these are increasingly mitigated by advances in GPU-accelerated algorithms. Under FCP conditions, MVS faces substantial challenges: wind-induced plant motion violates the static scene assumption underlying structure-from-motion algorithms (Biskup et al., 2007), variable illumination causes feature-matching failures (Paturkar et al., 2019), and dense canopy occlusion limits individual plant resolution. Consequently, FCP applications typically achieve centimeter-level accuracy (R² = 0.78–0.99 for canopy traits; Klodt et al., 2015; Kim et al., 2021) and focus on plot-level rather than organ-level phenotyping. The recurring trade-off involves data quality versus acquisition flexibility: CCP enables controlled, high-resolution imaging at the cost of ecological validity, whereas FCP captures field-relevant phenotypes with reduced geometric precision and increased susceptibility to environmental artifacts.

### 2.3 Time-of-flight cameras

Time-of-flight (ToF) cameras represent a cutting-edge imaging and distance measurement technology. Unlike traditional cameras that capture 2D images based on color and intensity, ToF cameras measures the time it takes for light to travel from the camera to the object and back. This allows them to create depth maps and 3D representations of the scene (Gokturk et al., 2004; Keller and Kolb, 2009). Two primary approaches are used in time-of-flight (ToF) systems, each offering unique advantages for different applications. Direct ToF (dToF) cameras emit a brief light pulse lasting only a few nanoseconds and directly measure the time delay between the emission of the light pulse and its reflection from an object, calculating the distance based on the speed of light. Indirect ToF (iToF) cameras, on the other hand, emit continuously modulated light pulses (diffuse laser illumination) and measure the phase shift in the frequency of the reflected light to determine the distance to an object (Li, 2014; Padmanabhan et al., 2019). This method is beneficial for measuring the entire scene of objects close to the camera and allows iToF cameras to achieve higher frame rates. ToF cameras offer several advantages, including high frame rates, real-time depth information capture, and robustness under various lighting conditions. These characteristics make them suitable for multiple applications, including gesture recognition, industrial automation, augmented reality, and robotics.

The versatility and precision of ToF cameras have spurred interest in their application in the agricultural industry. In particular, they hold significant potential for plant phenotyping by providing valuable data on plant structures, growth patterns, and health statuses. The depth information captured by ToF cameras allows researchers to create detailed 3D models of plants, enabling precise measurements of plant height, leaf area, and biomass.

Researchers have integrated ToF cameras into phenotyping platforms to automate the collection of morphological data. Several studies in the literature have explored the application of time-of-flight (ToF) cameras in plant phenotyping under CCP and FCP conditions, demonstrating their usefulness and addressing various challenges associated with depth imaging.

For CCP applications, Song et al. (2011) and Alenya et al. (2011) focused on enhancing depth estimation and 3D modeling by integrating ToF cameras with other imaging techniques. Song et al. (2011) combined stereo and ToF images to estimate dense depth maps for automated plant phenotyping. They developed a geometric approach to transform the ToF depth information for stereo imaging, focusing on challenging plant images captured in a glasshouse environment. Despite their success, they faced challenges with the reliability of ToF data under dynamic lighting conditions and the difficulty in obtaining accurate pixel-by-pixel depth data. Alenya et al. (2011), on the other hand, used ToF cameras in conjunction with color data for robotic plant measurements. By combining hierarchical color segmentation with quadratic surface fitting using ToF depth data, they successfully interpolated depth maps that closely matched the original scenes. However, they encountered difficulties in accurately segmenting overlapping leaves and managing occlusions, which are common issues in dense plant canopies.

In a comparative analysis, Kazmi et al. (2014) examined the performance of ToF cameras and stereo vision sensors under various illumination conditions. They tested three ToF cameras (PMD CamBoard, CamCube—pmd Group of Companies, Siegen, Germany, and SwissRanger SR4000—MESA Imaging AG, Technoparkstrasse 1, 8005 Zurich) against stereo correspondence algorithms, assessing their efficacy in indoor and outdoor settings. They found that ToF cameras had varying performances based on the lighting conditions, with the PMD CamCube excelling in sunlight. Nonetheless, ToF cameras struggle with ambient light interference, low resolution, and limited range. Kazmi et al. (2014) also proposed methods to enhance the dynamic range of ToF cameras, highlighting the strengths and limitations of both technologies in-depth imaging. This study underscores the need for improved algorithms to mitigate the effects of changing light conditions and enhance depth accuracy.

Focusing on low-cost solutions, Cao et al. (2017) developed a ToF-based depth imaging system for phenotyping plants, explicitly targeting branch and seedpod detection. Using ToF cameras to capture 3D videos and images, they created 3D models to estimate the plant characteristics. Their findings underscored the potential of low-cost ToF cameras for efficient and high-throughput plant phenotyping, particularly for estimating biomass and crop yield. However, they noted challenges in processing large volumes of data and ensuring consistent accuracy across species and growth stages.

Lately, Yang and Cho (2021) and Ma et al. (2022) further advanced the application of ToF technology by combining it with other sensors for more precise phenotypic analysis. Yang and Cho (2021) integrated a Kinect v2 depth sensor with an RGB camera to achieve high-resolution 3D crop reconstruction. Their system and algorithms enabled the accurate reconstruction and automatic analysis of phenotypic indices for red pepper plants, demonstrating high accuracy with an error margin of approximately 5 mm or less. Despite their success, they faced issues related to sensor calibration and the complexity of processing high-resolution data sets. Similarly, Ma et al. (2022) proposed a method for the automatic extraction of phenotypic traits from soybean canopies using 3D point cloud data acquired with a Kinect sensor. They developed a process for segmenting individual plants and calculating traits, such as plant height and leaf area index, and reported a high correlation between the estimated and manually measured values. However, they encountered difficulties in dealing with plant movement and variations in plant structure, which can affect the accuracy of trait measurements.

Likewise, several studies have explored the application of ToF cameras for plant phenotyping under FCP conditions, demonstrating both the utility and challenges of this technology in dynamic outdoor environments. Klose et al. (2009) and Moller et al. (2009) investigated the usability of 3D ToF cameras for automatic plant phenotyping and plant height measurements in field trials, respectively. Klose et al. (2009) focused on evaluating the performance of ToF cameras under varying outdoor conditions, such as direct sunlight, speed, humidity, and dust. They analyzed the color dependence, noise level, and depth resolution of cameras to determine their suitability for phenotyping applications. Moller et al. (2009) applied ToF cameras to measure the height of triticale in field trials. Their system utilized modulated light sources to calculate the distances for each pixel, enabling measurements while driving through test plots. The system achieved height estimation accuracy with a mean error of approximately 10%–14% relative to manual measurements, although the performance varied with growth stage and environmental conditions. Despite achieving good results, challenges include managing environmental influences and ensuring the accuracy of height measurements during different growth stages.

Ruckelshausen et al. (2009) and Busemeyer et al. (2010) extended the use of ToF technology to more sophisticated phenotyping platforms. Ruckelshausen et al. (2009) developed BoniRob, an autonomous field robot with multi-sensor systems, including ToF cameras, for individual plant phenotyping. This robot utilizes probabilistic robotics for navigation and multi-sensor fusion for accurate phenotypic measurements, emphasizing the importance of robustness and flexibility in field applications. Busemeyer et al. (2013) further enhanced BreedVision, a multi-sensor system integrated into a tractor for phenotyping high-density crop field plots, which they first developed (Busemeyer et al., 2010). This platform combines ToF cameras with other optical sensors to obtain comprehensive spectral and morphological data. They highlighted the importance of repeatability and robustness in sensor measurements, addressing challenges such as sensor calibration and data quality evaluation under field conditions.

Li and Tang (2017) proposed a low-cost 3D plant reconstruction system using a 2D camera and a 3D ToF camera. They focused on developing algorithms for the precise alignment of multiple 3D views, enabling accurate 3D reconstruction and morphological trait characterization of corn seedlings. Their system demonstrated promising accuracy and speed, although challenges included maintaining alignment precision and handling complex plant structures. This study underscores the potential of affordable and high-performance phenotyping systems to enhance high-throughput phenotyping in indoor and outdoor settings.

Owing to the rugged nature of the field, many researchers have adopted field-based phenotyping robots because of their significant potential in large-scale agricultural applications and have focused on developing economically viable robotic platforms. For instance, Young et al. (2019) developed a low-cost robot for energy sorghum phenotyping, achieving plant height measurement accuracy with R² = 0.90–0.99 and RMSE of 5 cm–8 cm, and stem diameter accuracy with R² = 0.85–0.92, demonstrating accurate plant height and stem width measurements over large areas. This system can be adapted for maize and other row crops, providing high spatial and temporal resolution data. Fan et al. (2022) presented a similar high-throughput phenotyping robot equipped with RGB-D cameras, achieving effective stem diameter measurements in challenging conditions of maize crop rows. Song et al. (2023) proposed a dynamic 3D data acquisition method using a consumer-grade RGB-D camera on a movable platform. This method efficiently collected RGB and depth images of crop canopies and achieved plant height estimation with R² = 0.94–0.99 and leaf area index correlation of R² = 0.90–0.96 across different maize growth stages. The system proved effective under various conditions, including different times of day and moving speeds, thus demonstrating its suitability for outdoor crop phenotyping.

Moreover, other studies have focused on optimizing methodologies and algorithms to fine-tune ToF applications in outdoor environments. This includes studies on poplar seedlings and maize plants that have demonstrated the capabilities of 3D ToF and RGB-D cameras in field phenotyping. For example, Hu et al. (2018) developed a method for measuring the leaf geometric characteristics of poplar seedlings using 3D visualization, demonstrating accurate measurements of leaf width, length, area, and inclination angle. Vázquez-Arellano et al. (2018) focused on maize plants and utilized 3D reconstruction methods with ToF cameras to produce detailed point clouds and successfully validated seedling positions with high accuracy. Similarly, Bao et al. (2019b) created an automated system for characterizing maize architectural traits, achieving satisfactory accuracies for plant height, leaf angle, and plant orientation, proving the robustness of the system despite occlusions caused by leaves.

Furthermore, environmental sensor data fusion with non-rigid plant reconstruction models has been proposed to allow for the quick visualization of the environmental conditions in which plants grow. According to Sampaio et al. (2021), fusion was performed through the colorization of the model regions, consistent with the sensor values at the heights where they were installed; in their proposed system, three height levels were selected. They experimented with three environmental sensors: temperature, humidity, and luminosity sensors. Their approach allows for accurate structural measurements and environmental mapping, enhancing crop efficiency and health evaluation.

In summary, ToF cameras present a distinctive performance profile characterized by high temporal resolution but moderate spatial accuracy in both environments. Under CCP conditions, ToF sensors achieve millimeter-level precision (<5 mm error; Yang and Cho, 2021) and enable real-time depth acquisition suitable for the dynamic monitoring of plant responses. Integration with RGB cameras facilitates simultaneous structural and color-based analyses, supporting applications from leaf segmentation to canopy LAI estimation (R² = 0.94; Ma et al., 2022). However, ambient light interference, multipath reflections, and relatively low spatial resolution compared to laser triangulation limit the suitability of this technology for fine-scale organ measurements. In FCP settings, ToF cameras face additional challenges from sunlight saturation, which degrades depth accuracy in outdoor conditions, and from the limited operational range of consumer-grade sensors (Kazmi et al., 2014). Despite these constraints, the technology has found successful field applications through integration with robotic platforms (e.g., BoniRob, BreedVision), where real-time acquisition speed compensates for reduced precision, achieving a 10%–14% mean error for canopy traits (Li and Tang, 2017; Young et al., 2019). The consistent pattern across studies indicates that ToF cameras are optimally positioned for applications prioritizing temporal frequency over spatial precision, time-series growth monitoring, dynamic response tracking, and real-time robotic guidance, rather than for high-accuracy static phenotyping.

### 2.4 Terrestrial Laser Scanning

**Figure 3:**
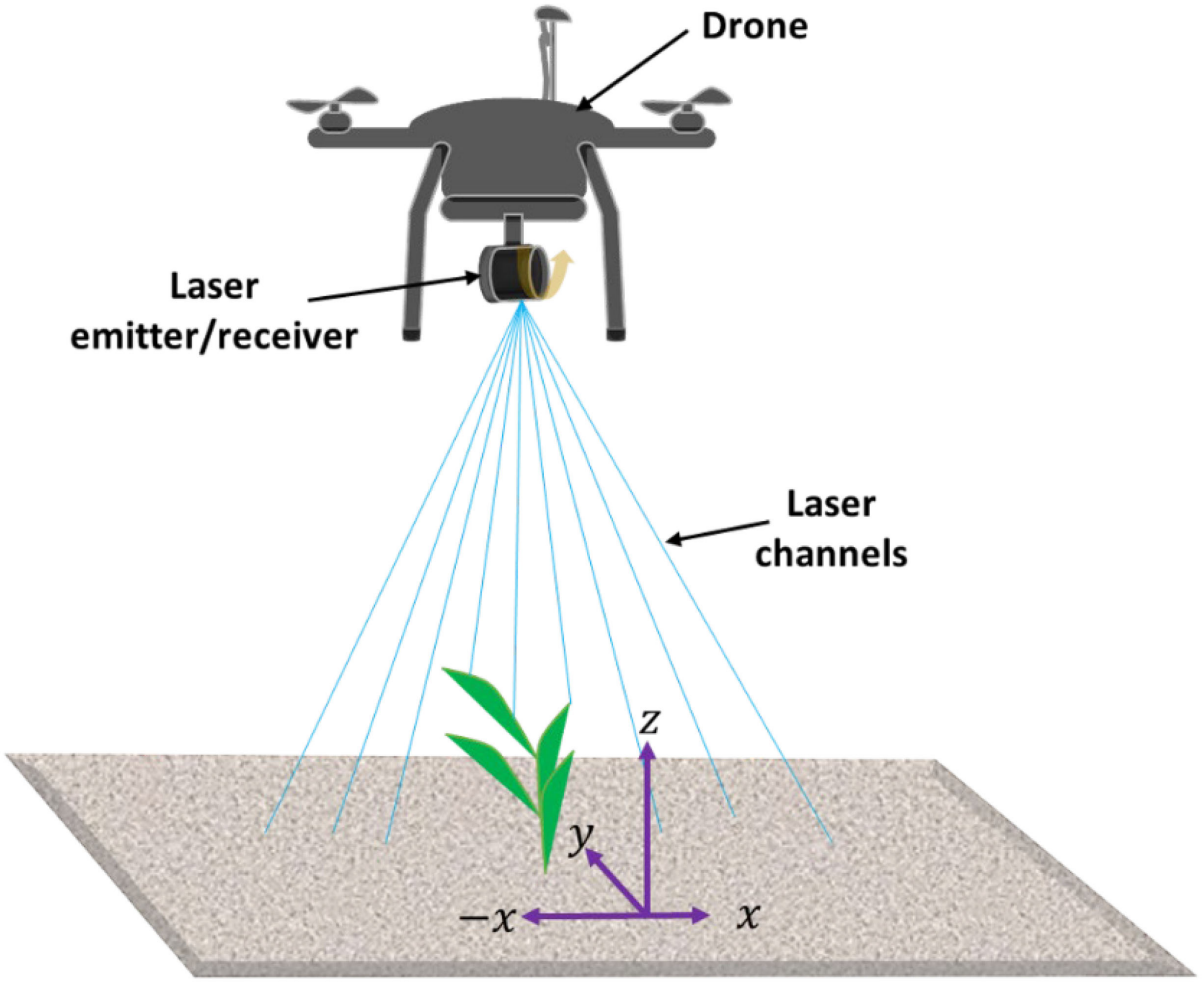
Principles of operation of a terrestrial laser scanner: Equipped with a multi-channel laser emitter for broader line-of-sight coverage and a rotating mechanism to scan a wider field of view.

Terrestrial Laser Scanning (TLS), also referred to as terrestrial light detection and ranging (LiDAR) or topographic LiDAR, is a remote sensing technology that captures precise three-dimensional (3D) information about objects and environments. Similar to 2D ToF cameras, TLS measures the time taken for emitted laser pulses to return after hitting a target. This time measurement calculates the distance to the target, enabling the creation of detailed 3D point clouds that accurately represent the scanned area. TLS devices consist of a laser emitter, a receiver, and, in most cases, a rotating mechanism to cover a large field of view (**Figure 3**). The laser emits rapid light pulses, and the time taken for each pulse to return to the receiver after reflecting off an object is recorded. These data were used to compute the distance, generating a 3D point cloud in which each point had specific coordinates (x, y, z). This process is similar to that of ToF cameras; however, TLS systems typically offer higher precision and range. This technology is primarily employed to rapidly acquire 3D information across a wide range of topographic and industrial objects. This enables the precise modeling and documentation of diverse subjects, including cultural heritage sites, bridges, plants, vehicles, coastal cliffs, highways, and traffic collision damage (Lemmens and Lemmens, 2011).

Notably, TLS sensors are implemented using various technologies, which can significantly influence their applicability under CCP and FCP conditions. Therefore, understanding the underlying concepts and classifications of this technology is essential. The first level of categorization was drawn from Van Genechten (2008). Their tutorial divided laser scanner technology into two main categories: static and dynamic. Static laser scanning involves keeping the scanner in a fixed position during data acquisition, offering high precision and a relatively high point density. While all static laser scanning can be considered terrestrial laser scanning, not all terrestrial laser scanning falls under the static category.

In contrast, dynamic laser scanning involves mounting the scanner on a mobile platform. This approach requires additional positioning systems, such as Inertial Navigation Systems (INS) and Global Positioning Systems (GPS), making the setup more complex and expensive. Dynamic laser scanning includes airborne laser scanning from an airplane, moving car, or unmanned aerial vehicle (UAV). Understanding these distinctions is crucial for appreciating the versatility and applications of TLS in various environments. Furthermore, Lemmens and Lemmens (2011) considered the measurement range of laser scanners to be one of the most important features of a TLS instrument as it significantly influences the types of applications for which they are suitable. The categorization is as follows: short-range laser scanners with a measurement range of up to 25 m, medium-range laser scanners that can measure distances of up to 250 m, and long-range laser scanners capable of measuring distances greater than 250 m. This classification further helps determine the appropriate scanner for specific tasks and environments based on the required measurement range.

Additionally, laser scanners can be categorized based on the underlying technology, which is crucial for determining suitable deployment environments (Colombo and Marana, 2010). These categories include: 1) pulse measurements, also known in TLS as 3D time-of-flight, where pulses are emitted, and their travel time to and from the object is measured; 2) phase shift, where waves are modulated in width or frequency, with width modulation being sensitive to sharp discontinuities in the shape or reflectance of the object, and frequency modulation providing reliable measurements even when the return energy is low; 3) optical triangulation, used for short-range applications and small objects; and 4) interferometry, which offers very high precision and is typically used in indoor industrial metrology. Phase-shift and pulse measurements are commonly utilized in TLS systems for outdoor applications. Understanding these categories and operational principles of laser scanning technologies sets the stage for exploring their practical applications in plant phenotyping.

In addition to classification by measurement principle, LiDAR systems for plant phenotyping can be categorized by deployment platform, each offering distinct trade-offs between spatial resolution, coverage, and operational complexity (Zhu et al., 2021; Jin et al., 2021). Terrestrial LiDAR (TLS), operated from fixed tripod positions or ground-based mobile platforms, achieves the highest point densities (typically 100 points/m²–10,000 points/m² at close range) and is optimal for the detailed structural characterization of individual plants or small plots. However, fixed TLS requires multiple scan positions to minimize occlusions, limiting the throughput for large-scale phenotyping.

Mobile terrestrial LiDAR (MLS), including vehicle-mounted, backpack, and handheld configurations, addresses the throughput limitations of static TLS while maintaining high point densities (typically 50 points/m²–500 points/m²). Backpack LiDAR systems, such as those described by Zhu et al. (2021), can phenotype hundreds of field plots per day, achieving a height estimation RMSE of 5–6 cm with sufficient point density for plot-level trait extraction. The integration of simultaneous localization and mapping (SLAM) algorithms enables continuous data acquisition without the need for external positioning references in certain systems.

Airborne LiDAR (ALS), deployed from manned aircraft or UAVs, provides the largest spatial coverage but at reduced point densities (typically 1 points/m²–50 points/m² for UAV-LiDAR and <1 point/m² for aircraft-mounted systems). UAV-LiDAR has emerged as a practical compromise, offering field-scale coverage with point densities that are sufficient for canopy-level trait extraction (Harkel et al., 2020). However, UAV payload constraints limit the sensor quality compared to terrestrial systems, and the regulatory requirements for larger UAVs add operational complexity.

Furthermore, the minimum point density required varies substantially according to the target trait. Canopy-level traits (height, cover, and volume) can be reliably extracted from point clouds with densities as low as 10 points/m²–50 points/m² (Madec et al., 2017), whereas organ-level traits (leaf dimensions and stem diameter) typically require densities exceeding 500 points/m² (Paulus, 2019). This relationship between point density and achievable trait resolution explains the continued role of high-density TLS in detailed phenotyping, despite the throughput advantages of airborne systems. For breeding applications focused on canopy-level selection traits, UAV-LiDAR provides adequate resolution. For physiological studies requiring organ-level measurements, terrestrial systems remain essential.

Until the late 2000s, the use of TLS in plant-related studies was limited to monitoring and modeling large forest tree species (Gorte and Pfeifer, 2004; Hosoi and Omasa, 2009; Preuksakarn et al., 2010). However, the focus is gradually shifting towards its application in crop monitoring and modeling under both CCP and FCP conditions. TLS has proven to be a powerful tool for plant phenotyping under FCP conditions, providing high-resolution and accurate data on plant structures and spatial distribution. The application of TLS in open-field environments presents different challenges and opportunities. Outdoor conditions introduce variability in lighting, weather, and plant interactions, affecting the quality of the collected data. The robustness of TLS technology allows for comprehensive assessments of large-field plant growth dynamics, health, and spatial distribution, providing insights that are crucial for improving crop management and breeding programs. Several studies have demonstrated the practical use of TLS for FCP conditions to perform growth monitoring (Dhami et al., 2020; Friedli et al., 2016; Yuan et al., 2018), health monitoring (Su et al., 2019), biomass estimation (Deery et al., 2020; Li et al., 2020; Pan et al., 2022) and yield prediction (Malambo et al., 2019). Beyond these primary applications, LiDAR-derived traits have been expanded to include canopy structural complexity indices (Zhu et al., 2021), leaf area index estimation through gap fraction analysis (Hosoi and Omasa, 2009), lodging severity quantification (Malambo et al., 2019), and temporal growth rate characterization through multi-date acquisitions (Jin et al., 2021). The ability to derive multiple traits from a single acquisition, including height, volume, surface area, and structural heterogeneity, positions LiDAR as a particularly efficient sensing modality for breeding programs that require comprehensive phenotypic characterization.

Despite its advantages for field phenotyping, LiDAR technology faces several challenges specific to FCP environments that can limit the data quality and trait extraction accuracy. Ground-canopy separation presents a fundamental difficulty in dense crop stands, where laser pulses may fail to penetrate to ground level, compromising the accuracy of height calculations that depend on digital terrain models (Zhu et al., 2021). Multi-return LiDAR systems partially address this issue by distinguishing the first and last returns; however, their performance is degraded in crops with overlapping canopy layers.

Wind-induced motion during scanning introduces noise and registration errors, which are particularly problematic for mobile platforms, where the scan duration may span several seconds per plot. Friedli et al. (2016) documented increased height estimation variance under windy conditions, recommending data acquisition during calm periods when feasible. Atmospheric conditions, including dust, fog, and precipitation, can attenuate laser returns and introduce spurious points, although active sensing is generally more robust to these factors than passive imaging techniques.

Canopy penetration varies with plant architecture and growth stage, affecting the structural characterization completeness. Erectophile canopies (erect leaves) typically permit greater laser penetration than planophile architectures, creating systematic differences in point cloud completeness among genotypes (Jin et al., 2021). The interaction between plant architecture and sensing geometry represents an often-overlooked source of measurement bias in comparative phenotyping studies. Finally, the computational burden of processing high-density LiDAR point clouds remains substantial, with datasets for large breeding trials potentially reaching terabytes and requiring specialized processing pipelines (Harandi et al., 2023).

Quantitative validation of TLS under field conditions demonstrated robust performance for canopy-level phenotyping. Yuan et al. (2018) compared ground-based LiDAR with UAV photogrammetry for wheat height estimation, finding LiDAR achieved superior accuracy (RMSE = 0.05 m, R² = 0.97) compared to UAV-SfM (RMSE = 0.09 m, R² = 0.91). Zhu et al. (2021) demonstrated that backpack-mounted LiDAR systems could phenotype wheat plots with a height estimation RMSE of 5 cm–6 cm while maintaining a throughput sufficient for large breeding trials (>500 plots per day). For biomass estimation, Deery et al. (2020) reported an R² of 0.86 between TLS-derived canopy volume and destructive biomass measurements in wheat plants. These results indicate that mobile TLS platforms can achieve field phenotyping accuracy approaching that of fixed-gantry systems while offering substantially greater flexibility and coverage.

In contrast, TLS has been used in CCP to enhance the accuracy and efficiency of phenotyping processes. For instance, Panjvani et al. (2019) developed a low-cost LiDAR-based 3D scanning system to estimate key leaf traits, such as length, width, and area. The LiDARPheno system used a LiDAR sensor interfaced with Arduino Uno and Raspberry Pi to create a cost-effective and user-friendly setup. This study demonstrated the potential of LiDAR to provide accurate phenotypic measurements, emphasizing its applicability in indoor settings, where traditional methods might fall short because of the complex structure of plants. Similarly, Patel et al. (2023) developed a deep learning-based approach to enhance individual plant organ segmentation and phenotyping under controlled scanning conditions using LiDAR point clouds. Furthermore, Wang et al. (2018) performed a comparative study of TLS and MVS reconstruction and concluded that TLS provided satisfactory point clouds for medium- and high-maize plants with acceptable efficiency. However, the results were not adequate for small maize plants. A more recent comprehensive review of TLS applications in crop management for precision agriculture can be found in Farhan et al. (2024).

In summary, TLS occupies a unique position among 3D phenotyping technologies, with its primary strengths more naturally aligned with FCP than with CCP applications. Under controlled conditions, TLS provides sub-millimeter to millimeter accuracy and enables detailed architectural measurements (R² >0.90 for organ traits; Wang et al., 2018; Thapa et al., 2018), but its advantages over simpler, lower-cost alternatives (laser triangulation, structured light) are limited when environmental control eliminates the need for long-range capability and lighting independence. In contrast, TLS demonstrates robust performance under field conditions, where its active illumination, independence from ambient light, and extended measurement range (up to 250+ m for long-range systems) provide decisive advantages. Field studies have consistently reported centimeter-level accuracy (RMSE = 5–6 cm for plant height; Yuan et al., 2018; Dhami et al., 2020) and strong correlations for biomass estimation (R² = 0.86; Deery et al., 2020). Recent innovations in mobile and backpack-mounted LiDAR systems (Zhu et al., 2021) have substantially improved field throughput while maintaining accuracy, thereby enabling large-scale phenotyping across hundreds of plots. Key limitations of FCP include challenges with ground-canopy separation in dense vegetation, wind-induced noise during scanning, and computational demands for processing high-density point clouds. The synthesis across studies reveals that TLS is optimally deployed for field-scale structural phenotyping, where environmental robustness and measurement range outweigh the cost and complexity considerations that favor simpler technologies in controlled environments.

### 2.5 Structured light approaches

**Figure 4:**
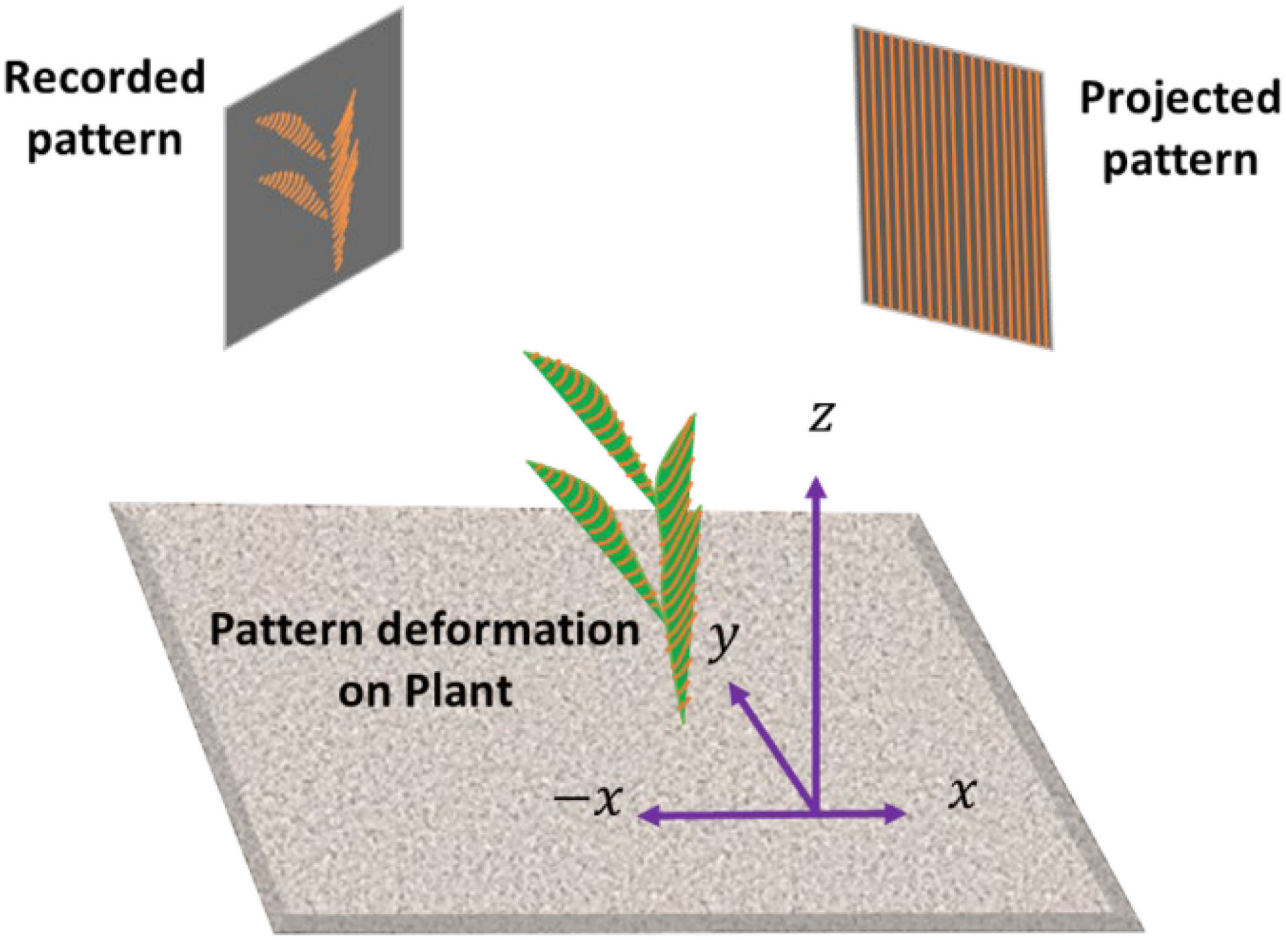
Structured light scanning mechanism: A projector emits a predetermined pattern, while a camera (or a pair of cameras) captures light deformation on the target object.

Structured light (SL) scanning is an advanced optical technique widely used in various fields for precise 3D surface measurements and imaging. This technology operates by projecting a series of light patterns, often in the form of grids or stripes, onto objects (Salvi et al., 2010). The deformation of these patterns when they interact with the object’s surface is captured by a single camera or a pair of cameras (**Figure 4**). By analyzing the captured images, complex algorithms can reconstruct the 3D geometry of an object with high accuracy and resolution.

Similar to laser triangulation and stereo vision discussed previously, the core principle behind SL scanning involves replacing one of the cameras in stereo vision with a projector and applying the triangulation method, where the positions of the light projector and cameras are fixed and known (Salvi et al., 2010). When a light pattern is projected onto an object, each point on the surface creates a unique deformation in the pattern, allowing the system to calculate the precise 3D coordinates of those points. This method offers several advantages, including high precision capable of capturing fine details (Wan et al., 2021; Zhou et al., 2021), rapid data acquisition suitable for dynamic environments (Wang and Zhu, 2024; Zhou et al., 2021), and non-contact measurement that ensures that delicate objects are not disturbed or damaged. Structured light scanning has been employed in diverse industries, such as manufacturing, healthcare, cultural heritage preservation, and entertainment. In manufacturing, it ensures quality control by providing detailed inspections of parts and assemblies (Javaid et al., 2021). In healthcare, it aids in creating accurate 3D models for prosthetics and surgical planning (Olesen et al., 2011). The cultural heritage sector uses it for the preservation and digital archiving of artifacts (Kantaros et al., 2023), whereas in entertainment, it enables realistic 3D modeling for visual effects and animations (Hieda, 2015). Readers may refer to Zhang (2018) for a more systematic review of 3D shape measurements using SL methods.

Over the past few decades, the agricultural sector has recognized the potential of SL scanning, particularly in 3D morphological plant phenotyping. The adoption of SL scanning in plant phenotyping has numerous benefits. It provides a detailed morphological analysis by creating high-resolution 3D models of plants, capturing intricate details of plant shape, size, and structure (Nguyen et al., 2015). Similar to other approaches, it enables dynamic growth monitoring, allowing continuous observation of plant development over time, which is crucial for studying dynamic processes. This technology can also facilitate stress response analysis by assessing the physical changes in plants under various stress conditions, such as drought, disease, or nutrient deficiency (Nam et al., 2014). Furthermore, it supports high-throughput analysis and can quickly examine large numbers of plants, thereby enhancing the efficiency of phenotyping processes.

SL scanning systems are commonly integrated into CCP environments, such as greenhouses and growth chambers, for practical implementation. For example, Nguyen et al. (2015) described a novel 3D indoor reconstruction system for plants that utilizes multiple high-resolution digital cameras, structured illumination, and computer vision techniques to enable non-destructive phenotyping of various crop plants, including cabbage, cucumber, and tomato. Nam et al. (2014) also demonstrated the potential of using SL to detect changes in growth responses to abiotic stress based on 3D leaf area analysis from the reconstructed point cloud.

In the case of FCP applications, there are limited studies reported in the literature; nevertheless, studies such as Rosell-Polo et al. (2015) provide a very detailed analysis of the capabilities of SL in precision agriculture. According to the authors, field tests demonstrated that these SL sensors effectively captured RGB-D point clouds for detailed 3D models, which can support site-specific phenotyping applications, including weed control. Rosell-Polo et al. (2015) reported that under controlled outdoor lighting (dawn/dusk conditions), structured light systems achieved canopy volume estimation with R² = 0.99, although the accuracy degraded substantially (>50% error increase) under direct sunlight conditions. Additionally, although sensor performance is limited under high ambient light, their affordability, high frame rate, and flexibility render them valuable for precision agriculture and outdoor conditions during dawn, dusk, or nighttime. The scarcity of quantitative FCP validation studies for structured light systems represents a notable research gap, as most published accuracy assessments are derived from controlled environments.

In summary, structured light technology exhibits the most pronounced performance disparity between the CCP and FCP environments among all the reviewed techniques. Under controlled conditions, SL systems achieve millimeter-level accuracy (<13 mm error; Nguyen et al., 2015) and high correlations for leaf area and stress response measurements (R² >0.9; Nam et al., 2014), benefiting from the ability to control ambient lighting that would otherwise interfere with the projected patterns. This technology excels in high-resolution surface reconstruction for detailed morphological analysis and supports applications from leaf area quantification to drought stress detection. However, the fundamental dependence on controlled lighting represents a critical limitation for field deployment, as high ambient light severely degrades pattern detection, effectively restricting outdoor operations to dawn, dusk, or nighttime periods (Rosell-Polo et al., 2015). This operational constraint, combined with calibration sensitivity and the requirement for a fixed projector-camera geometry, has limited FCP adoption despite the affordability of the technology relative to laser-based alternatives. The consistent finding across studies is that structured light offers an attractive cost-accuracy balance for indoor phenotyping but cannot currently serve as a general-purpose field solution. Future developments in high-power projection and ambient light rejection may expand the operational envelope; however, at present, the technology remains predominantly CCP-oriented, with only niche FCP applications under controlled lighting conditions.

### 2.6 3D light field cameras

Light-field (LF) cameras represent an innovative leap in imaging technology, offering capabilities that extend far beyond those of traditional photography. Unlike conventional cameras, which capture a two-dimensional representation of a scene, LF cameras record the amount of light that travels in every direction through every point in space (Jeon et al., 2015). This is achieved by capturing the light field, a concept rooted in physics that describes the intensity and direction of light rays in a given environment (Wu et al., 2017).

**Figure 5:**
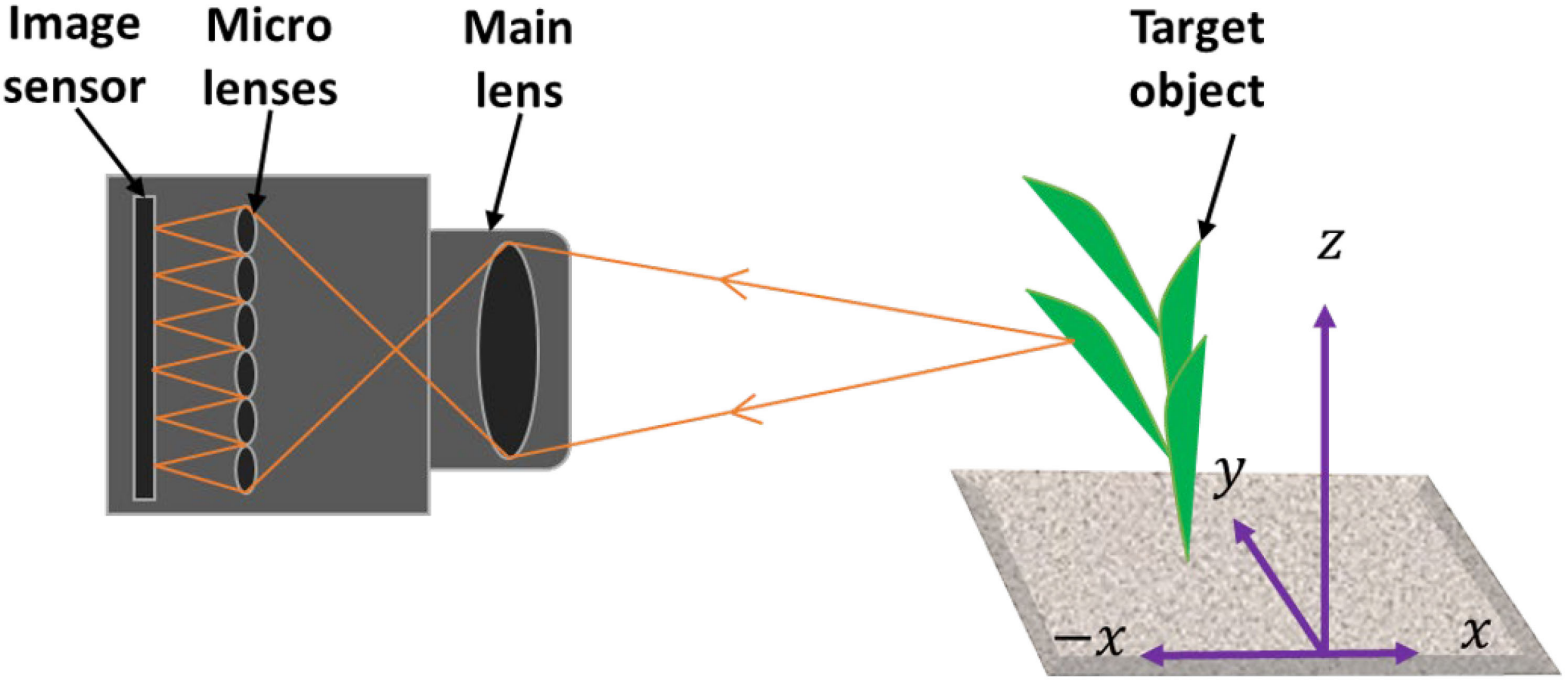
Operational principle of a light field camera: Features an array of micro-lenses positioned in front of the camera’s main lens.

The core of LF technology lies in its ability to capture both spatial and angular information of light rays. This is typically accomplished using an array of micro-lenses placed in front of the camera’s main lens (Wu et al., 2017). Each microlens captures light from different angles, allowing the camera to reconstruct the entire LF (**Figure 5**). This process results in a wealth of data that can be manipulated post-capture, enabling features such as refocusing, changing the depth of field, and creating 3D images from a single exposure (Jeon et al., 2015). In other words, the most significant advantage of LF cameras is their ability to refocus images after they have been captured. This feature is possible because the camera records light from multiple perspectives, allowing users to select different focal points during post-processing. Additionally, the depth information captured by LF cameras enables the creation of stereoscopic images, making it possible to render scenes in 3D and extract depth maps for various applications.

LF technology has primarily been applied in various fields, including virtual reality, computational photography, and industrial inspection. Its ability to capture and manipulate 3D information makes it particularly valuable in areas where precise spatial data are essential. For instance, in cinematography, LF cameras allow filmmakers to create immersive experiences by capturing scenes that can be navigated and refocused during post-production, offering unprecedented creative flexibility (Broxton et al., 2020).

Building on the technological foundation of light-field cameras, their application in agriculture, particularly in plant phenotyping, represents a promising frontier. In plant phenotyping, LF cameras can capture detailed 3D models of plants, enabling researchers to analyze various target traits and their growth patterns. The depth information provided by LF cameras is beneficial for assessing traits that are difficult to measure with traditional 2D imaging, such as leaf angle distribution and canopy structure (Polder and Hofstee, 2014). Moreover, the ability to refocus images allows for more accurate measurements of these traits, as researchers can adjust the focal plane to capture sharp images of specific plant parts (Schima et al., 2016).

According to Polder and Hofstee (2014), one of the key advantages of using LF cameras for plant phenotyping is the potential for automated large-scale data collection. By integrating LF cameras with machine learning algorithms, systems can be developed that automatically analyze plant traits from captured images, significantly reducing the time and effort required for phenotyping. This capability is especially valuable in breeding programs, where large populations of plants need to be evaluated for desirable traits.

Furthermore, Schima et al. (2016) underscored the potential of LF cameras as a powerful tool for on-site crop monitoring. This study evaluated a light-field camera system capable of capturing plant growth dynamics and traits in a field environment. The immunity of this technology to ambient conditions, such as varying light levels and environmental changes, makes it an effective tool for long-term plant monitoring, offering reliable performance across different settings. This makes light-field cameras particularly useful for large-scale in-field applications where traditional imaging systems might struggle with environmental variability. The integration of light-field cameras into real-time crop growth monitoring systems improves the spatial accuracy of trait measurements. This enhances the ability to track changes in plant morphology over time, providing valuable data for breeding programs and in precision agriculture. This study also highlighted the cost-effectiveness of these cameras in large-scale agricultural research, making them practical solutions for automated data collection across diverse crop types and environments.

While LF cameras hold significant potential for plant phenotyping, especially in capturing 3D spatial data and enabling automated large-scale trait analysis, their adaptation in this field has been limited by several technological and operational challenges. Schima et al. (2016) and Polder and Hofstee (2014) provided valuable insights into the current limitations of LF cameras, which likely explains their slower adoption in phenotyping practices.

A key limitation highlighted by Schima et al. (2016) is the limited depth resolution of LF cameras, particularly at long distances. For example, the Lytro LF exhibited accurate depth estimation only within a range of 10 cm–50 cm, which is insufficient for many field-based phenotyping tasks that require larger distances between the camera and plants. This short range significantly restricts the utility of LF cameras for large-scale plant height estimation or other growth monitoring tasks in tall crops or large-field plots. While newer models, such as the Lytro Illum, have improved sensor sizes, the small stereoscopic base of early models, such as the Lytro LF, makes them unsuitable for accurate measurements over larger distances. Furthermore, Schima et al. (2016) also report that cost remains a barrier, as achieving higher pixel resolution and depth accuracy would require more advanced (and expensive) cameras with larger sensors and improved microlens arrays.

Polder and Hofstee (2014) highlighted the additional technical challenges that arise when deploying LF cameras in greenhouse environments. One such issue is the complexity of calibration for accurate depth- and focus measurements. Over-saturation of pixels during calibration was found to disturb the proper calculation of depth and focus, which is a significant problem in environments where lighting is variable or difficult to control. Additionally, the fixed aperture setting of the camera (f/11) presented limitations in terms of image intensity control, particularly in combination with flash illumination, which is crucial for consistent daytime imaging in phenotyping environments. These constraints make it difficult to use LF cameras in practical phenotyping setups without significant modifications or expensive equipment.

Moreover, the limited field of view (FOV) of the LF camera is another significant drawback of phenotyping, as noted by Polder and Hofstee (2014). In their experiment, the LF camera had a maximum FOV of 50°, which was insufficient to capture entire plants, notably taller plants growing in greenhouse environments. This necessitates additional cameras or optical enhancements, such as mirrors, to achieve a wider FOV, further complicating the system design and increasing costs. This narrow FOV restricts the efficiency of image capture and data collection for large-scale phenotyping, rendering the technology less practical for high-throughput phenotyping.

The heavy computational requirements of LF cameras also present a significant challenge, as noted by Schima et al. (2016) and Polder and Hofstee (2014). The large file sizes of LF images (approximately 40 MB per image in Polder’s study) require substantial computing power to process depth and focus information. In robotic platforms, such as PhenoBot (Richardson et al., 2023), the power consumption of the computing systems required for LF processing was found to strain the battery resources of the robot, suggesting that a distributed setup (where images are captured on the robot and processed on a separate computer) is necessary to avoid battery depletion. This additional infrastructure increases the complexity and cost of the phenotyping system.

The quantitative validation of light-field cameras under field conditions remains extremely limited. Schima et al. (2016) reported plant height estimation with an average deviation error of 4.33 units (sensor-specific scale) under field conditions, representing substantially lower accuracy than achieved by MVS or TLS alternatives. The scarcity of quantitative FCP studies on light-field technology reflects both the relative immaturity of this technology for agricultural applications and the practical challenges of deploying these systems outside controlled environments. This gap in field validation represents a significant barrier to assessing the potential of this technology for routine phenotyping applications and should be addressed in future research.

In summary, light-field cameras represent an emerging technology with distinctive capabilities, particularly post-capture refocusing and single-exposure depth acquisition, but they have substantial limitations that have constrained their adoption in both CCP and FCP contexts. Under controlled conditions, LF cameras enable novel analytical approaches, including depth-based segmentation and variable focal plane analysis, for stem and leaf characterization (Polder and Hofstee, 2014). However, effective depth estimation is limited to short ranges (10 cm–50 cm; Schima et al., 2016), the narrow field of view necessitates multiple captures or optical enhancements for whole-plant imaging, and substantial computational resources are required for processing (file sizes approximately 40 MB per image). These constraints have prevented LF technology from competing with established approaches for routine CCP phenotyping, despite its unique capabilities. In FCP settings, additional challenges emerge: the short effective range limits the applicability to close-range ground-based systems, and the computational demands strain the mobile platform’s power budget (Richardson et al., 2023). Field validation has been limited, with reported accuracy (4.33 average deviation error for height; Schima et al., 2016) being below that achieved by more mature technologies. The synthesis across available studies suggests that light-field cameras currently occupy a specialized niche for applications requiring post-capture focal adjustment or depth-from-single-exposure capabilities but do not yet offer compelling advantages over MVS or ToF alternatives for general phenotyping tasks. Continued development of sensor resolution, depth range, and processing efficiency is required before LF technology can achieve broader adoption in plant phenotyping workflows.

## 3 Sensor mounting and carrier platforms

Effective 3D precision crop management relies not only on the sensors themselves but also on their strategic deployment. The mounting and carrying of sensors is a key factor in ensuring accurate and reliable data capture across various environments. Whether in controlled laboratory settings or challenging outdoor fields, the choice and/or design of mounting mechanisms and carrier platforms determines the quality and consistency of phenotypic data. Furthermore, 3D precision sensors naturally perceive the world within a given scope (i.e., in terms of visibility and distance) and thus require either the sensor or the target object to be moved and/or rotated to capture distant and obscured sides or objects. This section explores the critical role of sensor mounting and carrier platform. It offers insights into various options and their suitability across different agricultural contexts to optimize data collection and generate precise and actionable insights for crop management and improvement.

### 3.1 Sensor mounting

**Figure 6:**
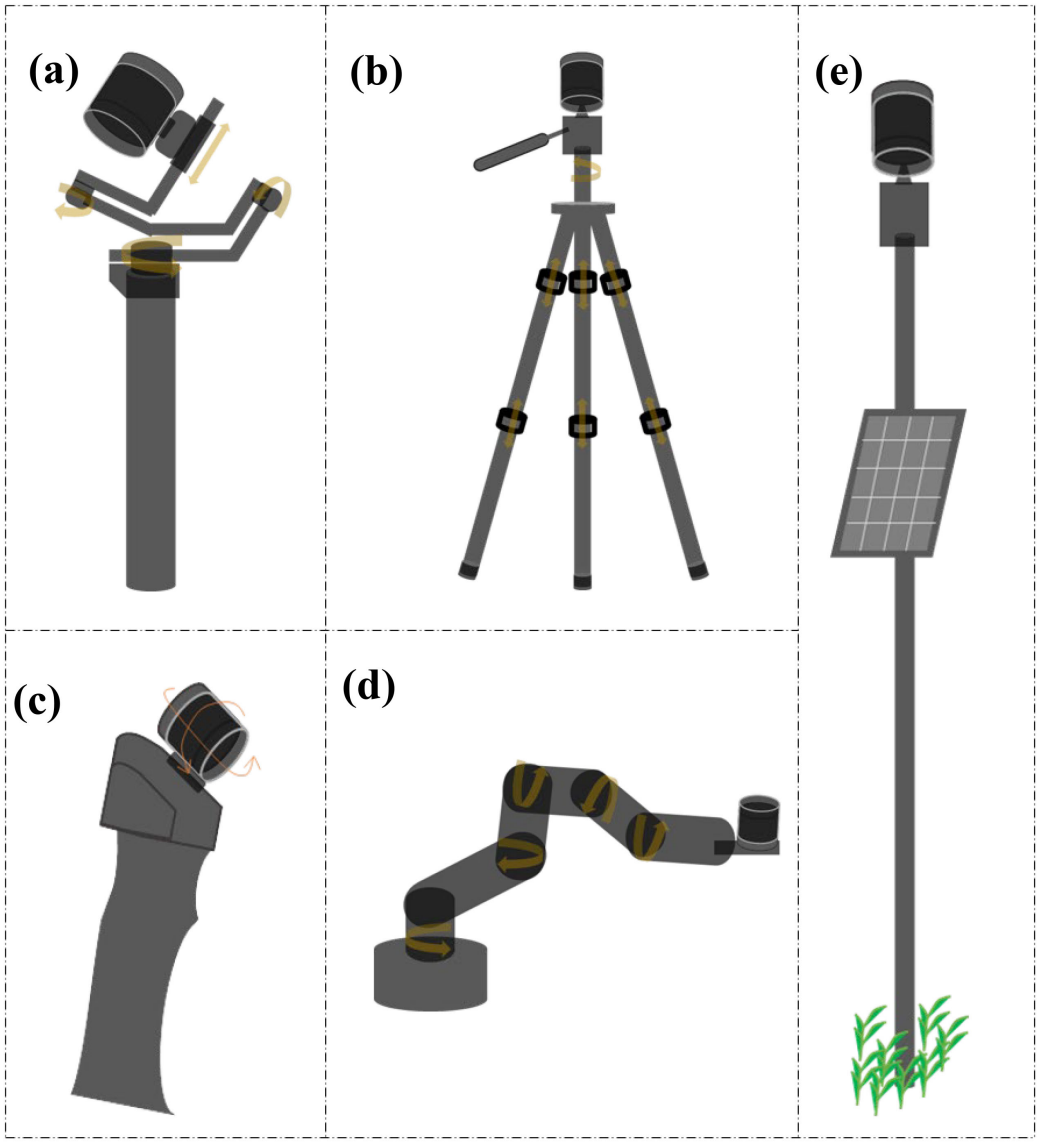
Common sensor mounting configurations in 3D phenotyping: **(A)** gimbal, **(B)** tripod stand, **(C)** handheld holder, **(D)** robotic arm, and **(E)** fixed post.

In this study, sensor mounting refers to the method or mechanism by which a sensor is physically positioned and secured to capture the data. Depending on the configuration, sensor mounting mechanisms must provide the desired stability, accuracy, and flexibility in different environments, whether in controlled laboratory settings or challenging field conditions. The choice of mounting method, whether a tripod, articulated arm, gimbal, or handheld setup, affects not only the precision of the data but also the efficiency and scope of the phenotyping process. This subsection introduces various sensor mounting mechanisms (**Figure 6**), examining their technical attributes and how they contribute to optimizing data capture for precise crop management and analysis.

#### 3.1.1 Tripods and fixed posts

Tripods and fixed posts are among the most commonly used mounting mechanisms for sensor deployment, particularly in controlled environments such as laboratories or greenhouses. These mechanisms provide a stable and stationary sensor platform that ensures consistent positioning over time. Tripods are adjustable in height and angle, allowing flexibility in sensor placement, which is crucial for capturing data at different stages of plant growth or from various angles. For instance, Alenya et al. (2011) used a tripod-mounted time-of-flight (ToF) camera to monitor maize plant growth in a greenhouse environment. The tripod ensured that the camera remained perfectly still, allowing for precise and repeatable 3D scans at multiple growth stages. According to the authors, this setup was crucial for analyzing the volumetric changes and structural development of the plant, as even minor shifts in the camera position could introduce significant errors in the 3D reconstructions. Fixed posts, often more robust and permanently installed, offer unparalleled stability, making them ideal for long-term monitoring in field conditions where a terrestrial laser scanner (TLS) can be employed to monitor the growth and structure of trees or taller crops.

Béland et al. (2014) used a fixed post to mount a TLS device for the 3D scanning of forest canopy structures. This provides a comprehensive view of tree architecture, capturing detailed data on branch and leaf distributions, which is critical for studying light interception and biomass estimation in forestry research. Both methods are beneficial for high-precision measurements, where the sensor movement is complex and/or may introduce errors or inconsistencies in the data.

#### 3.1.2 Articulated arm

Articulated arms are versatile mounting mechanisms that provide high sensor positioning and orientation flexibility. These arms can be adjusted autonomously in multiple directions and angles, allowing the sensors to be positioned precisely and moved dynamically from various perspectives during data collection without the need to reposition the plant. This is particularly advantageous when data need to be captured from multiple perspectives or when the sensor needs to be repositioned frequently without disturbing the plant or the surrounding environment (Atefi et al., 2021). Articulated arms are often used in laboratory settings for detailed phenotyping tasks, such as scanning specific plant structures or capturing images from various angles, and can be integrated with automated systems for high-throughput data acquisition. Paulus and Jens (2015) found that a robotic arm is crucial for enhancing the precision and flexibility of the laser scanning process used for phenotyping cereal plants. This system uses a robotic arm equipped with a laser scanner to capture 3D models of plant architecture, allowing for the high-resolution, non-invasive analysis of plant traits, such as canopy structure and stem alignment. The robotic arm provides precise control over the orientation and position of the sensor, facilitating the systematic scanning of plant surfaces and ensuring comprehensive coverage from multiple angles. This setup improves data consistency and accuracy, particularly in controlled environments where detailed plant morphology and growth patterns are monitored over time. The articulated arm’s ability to maintain consistent sensor positioning while allowing for dynamic adjustments makes it an invaluable tool for detailed morphological studies.

Moreover, recent advancements in quadruped robotics, particularly platforms such as Boston Dynamics’ Spot, have opened new possibilities for deploying articulated arms in field conditions (Lopes et al., 2023). The mobility and adaptability of quadruped robots make them ideal for navigating complex terrains, where traditional wheeled or stationary platforms are less effective. Equipping quadrupeds, such as Spot, with articulated sensor arms makes it possible to maneuver sensors around plants in the field, capturing fine-scale morphological data that are typically only accessible in controlled environments.

#### 3.1.3 Gimbal

Gimbals are advanced mounting mechanisms designed to stabilize sensors, even when the platform on which they are mounted is in motion (Singh et al., 2016). This technology is particularly beneficial in field conditions where drones or robotic platforms are used to carry sensors. The gimbal allows the sensor to maintain a steady orientation, compensating for movement or vibrations, which is critical for capturing clear and accurate data. Gimbals are commonly used in aerial phenotyping with drones, where maintaining a stable image or scan is essential despite the movement of the drone (Gašparović and Jurjević, 2017). This ensures high-quality data capture, which is crucial for aerial imaging, mapping, and monitoring crop health across large fields. Shi et al. (2016) demonstrated using a gimbal-stabilized photogrammetry system mounted on a UAV for high-throughput phenotyping in agricultural research. The gimbal stabilization was critical in maintaining the orientation of the photogrammetric camera, ensuring that the captured images were free from motion blur and other distortions. This precision is essential for accurately generating digital surface models (DSMs). This study focused on measuring plant height using DSMs generated from UAV-captured images. The gimbal-stabilized setup ensured that the height measurements were accurate and reliable, providing valuable data for precision agriculture practices. This approach enables efficient large-scale data collection across uneven terrains, significantly improving the process of high-throughput phenotyping compared with traditional ground-based methods.

Similarly, a recent review by Tanaka et al. (2024) highlighted the advantages of using UAV-mounted sensors with gimbals for crop phenotyping. The authors emphasized that gimbal-stabilized sensors provide high-resolution images and reduce motion artifacts, thereby improving the accuracy of phenotypic data.

#### 3.1.4 Handheld

Handheld mounting mechanisms offer the most flexibility but require manual operation, making them suitable for specific and targeted data collection tasks (Paulus, 2019). These setups are often used in field conditions where mobility and the ability to capture data from various locations quickly are essential (Zhu et al., 2021). Handheld sensors are beneficial for on-the-spot assessments, such as measuring plant height, leaf area, and other morphological traits. Although they lack the stability of fixed mounts or the precision of articulated arms, handheld devices allow researchers to collect data in areas that are difficult to reach or in situations where rapid assessment is required (Paulus, 2019). The portability of handheld mechanisms makes them ideal for exploratory research or situations in which the sensor must be moved frequently across different plants or plots (Paulus, 2019). Zermas et al. (2020) demonstrated the use of high-resolution RGB imagery collected with a handheld camera and UAV for 3D model processing in corn phenotyping. Their methodology involved structure from motion in reconstructing 3D canopies of small groups of corn plants, allowing for the automated extraction of phenotypic characteristics such as plant height, leaf area index (LAI), and individual leaf length. This approach provides accurate and frequent statistics for the in-season assessment of crop traits, enhancing the evaluation of crop performance and yield optimization. The handheld approach is particularly advantageous for capturing data from different parts of the plant, such as the lower canopy or areas that are not easily accessible by more extensive systems.

### 3.2 Sensor carrier platforms

**Figure 7:**
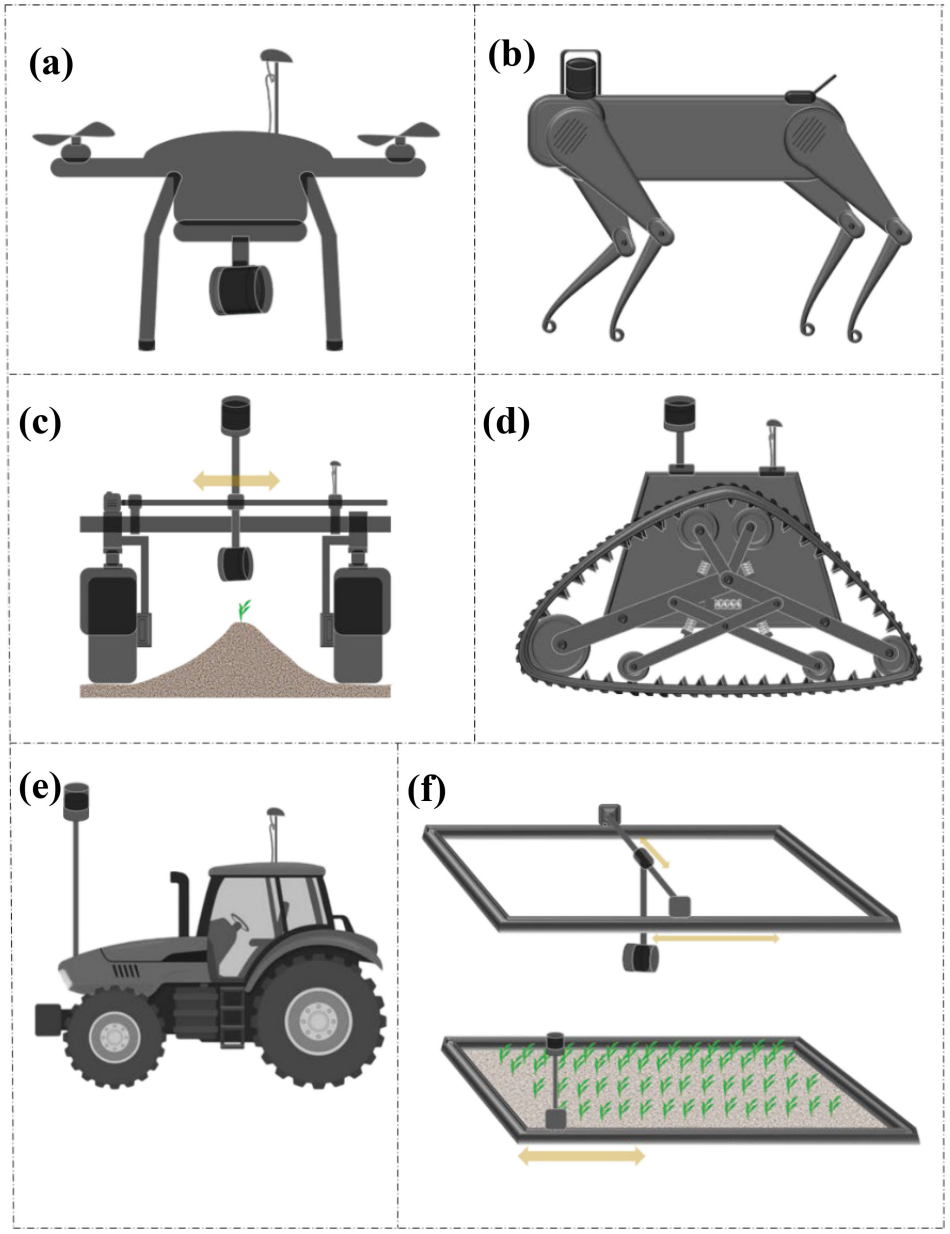
Common sensor platforms used in 3D phenotyping: **(A)** unmanned aerial vehicle (UAV), **(B)** quadruped robot, **(C)** wheeled robot, **(D)** treaded robot, **(E)** tractor, and **(F)** railed track (ground and overhead).

Likewise, sensor carrier platform refer to mobile systems used to transport and/or dynamically position sensors within different environments for data collection. In 3D precision crop management, the effective deployment and maneuvering of sensors across the target environment is vital for capturing high-quality phenotypic data. Sensor carrier platforms are critical in this process, providing the mobility, stability, and coverage required to obtain comprehensive datasets. These platforms range from ground-based systems, such as wheeled robots and tractors, to aerial platforms, such as drones, each offering unique advantages depending on the specific requirements of the phenotyping task. The choice of carrier platform impacts the resolution, efficiency, and scalability of data collection, making it a key factor in the design of any phenotyping strategy. Building upon sensor mounting, this subsection examines the various sensor carrier platforms (**Figure 7**) used in both CCP and FCP environments, highlighting their technical capabilities and applications in crop management.

#### 3.2.1 Drone (unmanned aerial vehicle, UAV)

In recent years, drones or UAVs have become one of the most widely used sensor carrier platforms in precision agriculture, enabling rapid large-scale data acquisition from the air. Equipped with 3D imaging sensors such as photogrammetry or LiDAR, drones can capture high-resolution data across extensive areas, providing detailed insights into plant canopy structure, biomass, and spatial variability. Their ability to cover large plots quickly and efficiently makes them particularly useful for field phenotyping in outdoor environments, especially when frequent data updates are necessary. For example, Zhu et al. (2023) demonstrated the use of extremely low-altitude UAV images for the quantitative estimation of organ-scale phenotypic parameters of field crops through 3D modeling. Their approach allowed for precise measurements of plant traits, such as leaf area and plant height, which are crucial for crop breeding and management practices. This study highlighted the potential of UAV technology to enhance the accuracy and efficiency of phenotyping processes. For a comprehensive review of drone-based imaging sensors, techniques, and applications in plant phenotyping, readers are referred to Gano et al. (2024), which provides an extensive analysis of the current state and future trends of UAV-based plant phenotyping.

#### 3.2.2 Wheeled and treaded robots

Wheeled and treaded (also known as tracked) robots are ground-based platforms used primarily in field environments for precision phenotyping applications. These autonomous or semi-autonomous systems can carry 3D imaging sensors at ground level, providing detailed spatial data on plant height, morphology, and structure. Wheeled robots are particularly effective for capturing close-range data, unlike aerial systems, especially for lower plant parts, such as stems and root zones. Iqbal et al. (2020a) developed a multipurpose autonomous differential drive mobile robot, MARIA, for plant phenotyping and soil sensing. The robot was designed to navigate autonomously using a global navigation satellite system (GNSS). It was fitted with an actuated LiDAR unit and depth camera to estimate plant morphological traits, such as volume and height. The robot’s three-degree-of-freedom manipulator allowed soil sensing and sampling, making it a versatile tool for phenotyping and soil analysis. Similarly, Xiang et al. (2023) demonstrated the use of a wheeled robot, PhenoBot, for field-based robotic leaf angle detection and characterization of maize plants using stereo vision and deep convolutional neural networks. The robot was equipped with PhenoStereo cameras to capture side-view images of maize plants, allowing precise measurements of leaf angles and other phenotypic traits. This approach significantly improved the efficiency and accuracy of phenotyping compared with traditional manual methods.

#### 3.2.3 Quadruped robotics

Quadruped robots are a newer class of sensor-carrier platforms designed to navigate complex and rugged terrains with greater flexibility and stability than wheeled or treaded robots (Katz et al., 2019; Lopes et al., 2023). These four-legged platforms can carry 3D sensors to capture detailed spatial data in environments where mobility is challenging, such as fields with dense vegetation and uneven ground. Their ability to traverse rough terrain makes them particularly valuable in outdoor agricultural settings, where precise data collection is required, but other robotic platforms may struggle. Lopes et al. (2023) discussed advancements in quadruped robotics, highlighting their applications in agricultural environments. According to their discussion, quadruped robots offer several advantages over traditional wheeled or treaded robots, including the ability to maintain stability on rough and uneven terrain, adaptability to different ground conditions, and enhanced maneuverability in tight spaces. The authors added that these robots are beneficial for phenotyping tasks in challenging field conditions, where other platforms may struggle. They can carry a variety of sensors, including LiDAR, cameras, and multispectral imaging systems, to collect high-resolution 3D crop data. Their study also detailed the design and development of a robotic arm specifically built to integrate with a quadruped robot for use in various agricultural applications. Quadruped robots can operate autonomously or be remotely controlled, making them versatile tools for detailed and accurate phenotyping in diverse environments (Lopes et al., 2023).

#### 3.2.4 Tractors

Tractors are a common platform for deploying sensors in large-scale agricultural settings, often serving as sensor carriers in precision farming. Mounted with 3D imaging systems, tractors enable data collection while performing other agricultural operations, such as planting or harvesting. For instance, Kise and Zhang (2008) developed a field-sensing system capable of performing 3D field mapping to measure crop height and volume and detect crop rows in 3D for reliable tractor guidance using a tractor-mounted stereo camera. The core of this dual-application field-sensing system is a stereovision-based mapping method. This method creates 3D crop structure maps by estimating the motion of a tractor-mounted stereo camera and progressively stitching the constituent stereo images. In a similar study, Sun et al. (2018) developed a high-throughput phenotyping system mounted on a tractor to scan plants from overhead using 2D LiDAR and RTK-GPS for precise spatial positioning. The system effectively reconstructs 3D models of crops by separating the ground plane and removing noise from weeds to generate clean 3D surface models of cotton plants. This setup allows for the measurement of key morphological traits, such as canopy height, projected canopy area, and plant volume, directly from the tractor, demonstrating its utility in large-scale agricultural settings. The ability to repeatedly scan entire fields over a growing season highlights the capability of tractor-mounted system for efficient and accurate data collection, which is essential for modern crop breeding and management practices.

#### 3.2.5 Ground and overhead rails

Ground and overhead rail systems are stationary or semi-stationary platforms used primarily in controlled environments, such as greenhouses or growth chambers. These systems allow sensors to move along fixed paths, capturing detailed 3D data over time without disturbing plants. Ground rails are typically used for lower- or mid-level plant phenotyping, whereas overhead rails offer a bird’s-eye view, which is ideal for capturing canopy structure and overall plant growth patterns. These systems are highly effective in environments that require continuous noninvasive monitoring, allowing consistent data capture with minimal human intervention. Li et al. (2023) utilized a hybrid (ground and overhead) design for a field rail-based phenotyping platform to collect high-throughput, time-series raw data of maize populations using LiDAR and RGB cameras. An earlier study by Vadez et al. (2015) utilized a similar novel arrangement in a greenhouse setting to combine 3D imaging and lysimetry for the high-throughput phenotyping of traits controlling the plant water budget. This system was designed to generate 3D crop structure maps, allowing for the accurate extraction of phenotypic traits.

Another notable approach is the use of a cable-suspended multi-sensor system to achieve similar novel concepts in rail-based systems, such as those in the studies by Kirchgessner et al. (2016) and Bai et al. (2019). These rail- and cable-based approaches enable precise measurements of plant height and volume, demonstrating the effectiveness of multi-source data fusion in improving the accuracy of phenotypic trait extraction.

### 3.3 Platform comparison and selection criteria

The preceding subsections have detailed individual mounting mechanisms and carrier platforms; however, selecting an appropriate platform for a given phenotyping application requires a systematic comparison across multiple criteria. **Table 2** synthesizes the key characteristics of the major platform categories to facilitate this selection process by comparing platforms across five dimensions: mobility and maneuverability, spatial coverage and throughput, measurement stability, cost considerations, and sensor compatibility.

**Table 2:** Platform comparison for 3D phenotyping applications.

Platform category	Mobility	Coverage (ha/day)	Stability	Capital cost	Operating cost	Compatible sensors	Best for
Fixed Gantry	None (fixed envelope)	<1 (but high temporal frequency)	Very High (<1 mm)	Very High ($500K–2M)	Low	All sensor types; multi-sensor arrays	Long-term plot monitoring, method development
Tractor-mounted	Moderate (row-constrained)	10–30	Medium (5–10 cm)	Medium ($20K–100K + vehicle)	Medium	LiDAR, RGB, multispectral, ToF	Large-field canopy phenotyping
Wheeled Robot	Moderate (terrain-limited)	5–15	Medium (2–5 cm)	Medium ($20K–80K)	Medium	LiDAR, RGB, ToF, structured light	Research plots, row crops
Tracked Robot	Good (rough terrain)	3–10	Medium (5–10 cm)	High ($50K–120K)	Medium	LiDAR, RGB, multispectral	Challenging terrain, orchards
Legged Robot	Very Good (all terrain)	2–8	Medium (5 cm–15 cm)	Very High ($75K–150K)	High	RGB, LiDAR (payload-limited)	Complex environments, demonstration
UAV (Consumer)	Excellent	50–100 (RGB)	Low (10 cm–20 cm)	Low ($1K–5K)	Low	RGB only	Rapid canopy assessment, NDVI
UAV (Professional)	Excellent	30–80 (multispectral)	Medium (5–15 cm)	Medium ($15K–50K)	Medium	RGB, LiDAR	Breeding trials, stress detection
UAV (Heavy-lift)	Excellent	10–30 (LiDAR)	Medium (5 cm–10 cm)	High ($50K–200K)	High	LiDAR, RGB	High-resolution structural phenotyping
Backpack/Handheld	Operator-dependent	1–5	Low (10 cm–30 cm)	Low-Medium ($5K–50K)	Low	LiDAR, RGB, handheld scanners	Targeted sampling, validation

Coverage estimates assume standard operating conditions and typical sensor configurations. Capital costs include platform and basic sensor payload; high-end sensors may substantially increase total system cost. Stability reflects typical effective positioning accuracy under operational conditions. Operating costs consider labor, consumables, and maintenance but exclude data processing. UAV operations subject to regulatory constraints that vary by jurisdiction.

#### 3.3.1 Mobility and maneuverability

Platform mobility fundamentally constrains the types of environment and crops that can be phenotyped. Fixed gantry systems offer no mobility but provide precisely controlled sensor positioning within their operational envelope, making them optimal for repeated measurements of the same experimental plots over time. Ground-based mobile platforms (tractors, wheeled robots, and tracked vehicles) provide moderate mobility, which is constrained by row spacing, soil conditions, and crop height. Legged robots offer superior terrain adaptability but at a substantially higher cost and complexity. UAV platforms provide maximum mobility and can access crops at any growth stage, although flight time limitations (typically 20 min–40 min) constrain single-mission coverage.

#### 3.3.2 Spatial coverage and throughput

Throughput requirements vary dramatically between breeding trials (requiring the phenotyping of thousands of plots) and physiological studies (requiring the detailed characterization of individual plants). UAV platforms achieve the highest throughput for canopy-level traits, capable of covering 50 ha–100 ha per day with RGB photogrammetry or 10 ha–30 ha with heavier LiDAR payloads (Shi et al., 2016). Ground-based mobile platforms achieve intermediate throughput (5 ha–20 ha per day, depending on driving speed and row spacing) while maintaining a higher spatial resolution. Fixed gantry systems have inherently limited coverage but enable the highest temporal resolution through the automated and repeated scanning of the same plots.

#### 3.3.3 Measurement stability

Stability directly affects the achievable measurement precision. Fixed gantry systems provide the highest stability, with a sensor positioning repeatability typically below 1 mm. Ground-based platforms introduce vibration and position uncertainty that can be partially compensated for through gimbal stabilization and RTK-GNSS positioning, achieving effective stability of 1 cm–5 cm. UAV platforms face the greatest stability challenges due to wind effects, GPS drift, and gimbal limitations, with effective positioning stability typically 5 cm–20 cm, depending on the conditions and equipment quality.

#### 3.3.4 Cost considerations

The platform costs span several orders of magnitude. Consumer UAVs with integrated RGB cameras represent the lowest-cost entry point (<$2,000), while research-grade UAV-LiDAR systems range from $50,000 to $200,000. Ground-based robotic platforms range from $20,000 for simple wheeled systems to $100,000+ for advanced legged robots. Fixed gantry systems represent the highest capital investment ($500,000–$2,000,000 for field-scale installations) but offer the lowest per-measurement operational costs for long-term studies. Importantly, the initial platform cost often represents a minority of the total phenotyping costs when labor, data processing, and maintenance are considered.

#### 3.3.5 Sensor compatibility

Platform payload capacity constrains the sensor options. UAVs face the most severe limitations, with consumer-grade systems (<5 kg payload) restricted to RGB cameras and lightweight multispectral sensors, whereas larger UAVs (5 kg–15 kg payload) can accommodate LiDAR or hyperspectral sensors. Ground-based platforms typically support all sensor types without significant payload constraints. Fixed gantry systems offer maximum flexibility for multi-sensor integration, commonly deploying arrays of RGB, hyperspectral, thermal, and LiDAR sensors simultaneously.

The optimal platform choice depends on the specific balance of these factors for each application of interest. UAV-based systems offer the best cost-effectiveness for large-scale breeding programs that prioritize throughput over precision. For detailed physiological studies requiring organ-level measurements, ground-based systems or fixed gantries provide the necessary precision. For phenotyping in challenging environments (sloped terrain, young crops, post-lodging), legged robots or handheld systems may be the only viable solutions.

## 4 Geometry phenotypes

**Figure 8:**
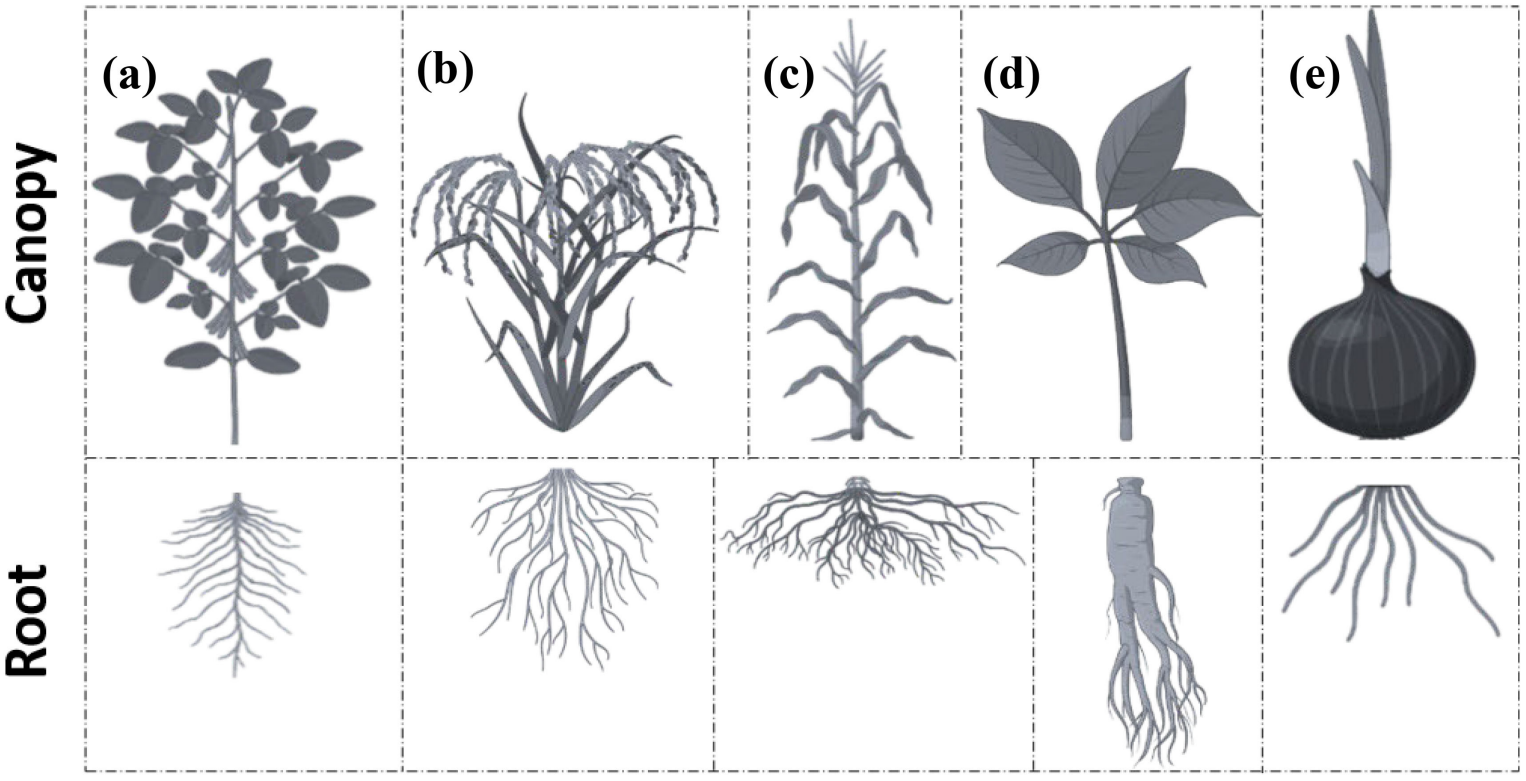
Variation in canopy and root architecture among common crop plants: **(A)** soybean, **(B)** rice, **(C)** corn, **(D)** ginseng, and **(E)** onion.

Selecting appropriate phenotypic traits is critical for leveraging modern sensor technology in both CCP and FCP phenotyping environments. The target end users of this information, including breeders and farmers, may require different, yet accurate and reliable data to assess and/or inform decisions on crop improvement and management strategies. This section reviews the key morphological and geometric traits of crops which are divided into two main categories: Canopy Architecture and Root Architecture (**Figure 8**). Each architectural characteristic is discussed in terms of its relevance, measurability, and challenges posed by CCP and FCP environments.

### 4.1 Canopy architecture

Canopy architecture refers to the spatial configuration of a plant’s aboveground organs, encompassing traits such as plant height, tillering, leaf area index, and the overall arrangement of leaves and branches (Fageria et al., 2006). These traits play a pivotal role in determining how effectively a crop intercepts light, utilizes resources, and withstands environmental stresses, which are critical for optimizing crop yields and efficiency. For breeders and farmers, understanding and optimizing canopy architecture is essential for improving genotype performance and enabling precision crop management.

The architecture of a plant(s) canopy is intrinsically linked to its ability to intercept solar radiation, which drives photosynthesis and ultimately determines crop productivity (Zegada-Lizarazu et al., 2012). According to Fageria et al. (2006), key traits such as plant height, leaf orientation, and tillering influence the distribution of light within the canopy, thereby affecting photosynthetic efficiency and resource use. For instance, a canopy’s stem characteristics, which include stem height, branching pattern, internode length, and stem diameter, are crucial for determining a plant’s growth, stability, and overall productivity. Taller plants with robust stems and erect leaves capture more sunlight, particularly in dense planting conditions, where lower leaves may otherwise be shaded. This increased light interception is vital for photosynthetic efficiency, driving better crop performance (Fageria et al., 2006; Evans, 2013). However, excessive stem height can lead to a higher risk of lodging, where plants may bend or collapse under their weight or due to external forces such as wind, significantly reducing crop yield and quality. In contrast, shorter plants with compact canopies may be preferred in environments prone to lodging because they are less likely to be damaged by strong winds or heavy rain. Similarly, a well-structured branching and tillering pattern enhances light penetration throughout the canopy, further promoting plant productivity. Conversely, excessive branching and/or tillering can lead to self-shading, which reduces light availability to the lower leaves and potentially hinders growth. Moreover, after panicle development in most cereal crops, competition for photoassimilates begins between panicles and tillers (Fageria et al., 2006).

Internode length and stem diameter also play key roles in the canopy architecture. Shorter internodes result in a more compact plant structure, which can be advantageous in environments where space is limited or where plants must resist lodging (Dahiya et al., 2018). However, longer internodes might improve light capture and increase lodging risk. A thicker stem provides greater mechanical support, reducing the risk of lodging and enabling the plant to support larger reproductive structures, such as fruits or grain heads. Additionally, stem diameter is associated with the plant’s capacity for nutrient and water transport, which is critical for sustaining growth and development under varying environmental conditions (Fageria et al., 2006).

Likewise, a canopy’s leaf characteristics, including leaf erectness (and/or angle), length, width, and thickness, play crucial roles in determining the overall yield potential of plant species or cultivars. Erect leaf orientation is critical, as it allows for greater light penetration and a more even distribution of sunlight within the crop canopy, thereby enhancing photosynthetic efficiency and increasing yield. This trait is often associated with higher-yielding varieties, as erect leaves reduce shading on lower leaves, enabling a more effective use of light (Fageria et al., 2006). Fageria et al. (2006) also argue that leaf thickness, which correlates with higher chlorophyll density per unit area, is linked to increased photosynthetic capacity and long-term gains in crop productivity. The size and angle of leaves are also significant; shorter, more erect leaves tend to distribute light more evenly and are less prone to drooping, which can reduce photosynthetic efficiency in taller cultivars than in shorter ones.

Although less variable than length, leaf width also contributes to yield by influencing the distribution of leaves within the canopy of the plant. Narrow leaves are generally preferred because they allow for more uniform light distribution, minimizing shading, and maximizing photosynthesis across the plant. Other important but non-morphological leaf characteristics include toughness, which is essential for preventing damage from wind and rain; color, an important indicator of plant health and nutrient status, with darker green leaves typically reflecting higher chlorophyll content and greater photosynthetic activity and senescence, the process of leaf aging that impacts the duration of photosynthetic activity. Early or rapid senescence can significantly reduce crop productivity by decreasing the photosynthetically active leaf area before the plant reaches its full yield potential (Fageria et al., 2006).

### 4.2 Root architecture

Root architecture, which refers to the spatial configuration of a plant’s root system, is a critical aspect of crop morphology that significantly influences the physiological aspects of plant growth and yield. The architecture includes key traits such as root length, diameter, surface area, and the distribution of root hairs, which collectively determine the plant’s ability to absorb water and nutrients from the soil. Both genetic factors and environmental conditions, such as soil type, moisture levels, and nutrient availability, shape the complexity and dynamics of root systems. For example, the effectiveness of a root system in nutrient uptake, particularly for relatively immobile nutrients such as phosphorus, is heavily dependent on the root’s surface area and its ability to efficiently explore soil volume (Takahashi and Pradal, 2021).

The root system plays a multifaceted role in supporting the plant by anchoring it, providing mechanical stability, and facilitating the absorption and transport of water, nutrients, and growth hormones to the shoots. Root architecture is also vital for plant responses to environmental stresses, such as drought or nutrient deficiencies. Plants with well-developed root systems are typically more resilient, as they can access deeper soil layers where water and nutrients are more abundant (Fageria et al., 2006). Despite its importance, root architecture has historically been studied less than aboveground plant structures because of the challenges involved in accessing and analyzing roots *in situ*. A more detailed discussion of root phenotyping and approaches can be found in Lynch (2022); Takahashi and Pradal (2021), and Wasaya et al. (2018).

### 4.3 Technology-trait suitability mapping

The selection of an appropriate 3D sensing technology for phenotyping specific architectural traits depends on multiple factors, including the spatial scale of measurement (organ, plant, canopy, or population level), required measurement precision, operational environment (CCP *vs*. FCP), and practical constraints, including cost, throughput, and technical expertise. This subsection provides explicit guidance for matching the sensing technologies reviewed in Section 2 with the phenotypic traits discussed above, addressing a critical decision point for researchers and practitioners designing phenotyping workflows.

#### 4.3.1 Scale-dependent technology selection

Phenotypic traits can be conceptualized across a hierarchy of spatial scales, each requiring different sensing approaches:

*Organ-level traits* (leaf dimensions, leaf angle, petal thickness, and internode length) require high spatial resolution and typically benefit from close-range sensing. Laser triangulation (LTS) is optimal for this scale in CCP environments, achieving micrometer-level precision that is suitable for detecting subtle morphological differences (Paulus et al., 2014). Structured light (SL) offers a lower-cost alternative with slightly reduced precision but faster acquisition times (Nam et al., 2014). For FCP applications at this scale, sensing options are limited; high-resolution MVS from ground-based platforms or ToF cameras integrated with robotic systems offer the most practical solutions, although with reduced accuracy compared to CCP alternatives.

*Plant-level traits* (plant height, stem diameter, branching pattern, and tiller count) are the most common phenotyping targets and are accessible to a broader range of technologies. In the CCP, all six reviewed technologies can address this scale, with the MVS offering the best balance of accuracy, cost, and throughput. In FCP, TLS (particularly mobile and backpack configurations) and MVS (both ground- and UAV-based) provide practical solutions with centimeter-level accuracy that is sufficient for breeding applications.

*Canopy-level traits* (canopy height, canopy cover, LAI, and canopy volume) require the coverage of larger areas and benefit from elevated sensing platforms. UAV-based MVS and LiDAR are the dominant technologies for FCP at this scale, capable of phenotyping hundreds of plots per day with an accuracy adequate for genetic analysis (R² >0.85 for height; Madec et al., 2017). Ground-based TLS can achieve higher precision but with reduced throughput. In the CCP, gantry-mounted LTS systems (e.g., Field Scanalyzer) combine the advantages of a controlled sensing geometry with plot-level coverage.

*Population-level traits* (spatial distribution patterns, lodging assessment and growth uniformity) require the largest spatial coverage and are predominantly the domain of UAV-based sensing. At this scale, MVS and LiDAR from UAV platforms provide the only practical solutions for both CCP and FCP, although the distinction between these environments becomes less meaningful when assessing population-scale phenomena.

#### 4.3.2 Technology-trait suitability matrix

**Table 3** provides a structured mapping of the technology suitability for specific phenotypic traits across CCP and FCP environments. Suitability ratings reflect the synthesis of published validation studies, considering both achievable accuracy and practical deployment.

**Table 3:** Technology-trait suitability matrix for 3D plant phenotyping.

Trait category	Specific trait	LTS	MVS	ToF	TLS	SL	LF	Optimal scale	CCP suitability	FCP suitability
Canopy architecture traits										
Stem traits	Plant height	••○	•••	••○	•••	••○	•○○	Plant/Canopy	High	High
	Stem diameter	•••	••○	•○○	••○	••○	•○○	Organ/Plant	High	Medium
	Internode length	•••	••○	•○○	••○	••○	•○○	Organ	High	Low
	Branching pattern	••○	•••	•○○	••○	•○○	•○○	Plant	High	Medium
	Tiller count	••○	•••	••○	••○	•○○	•○○	Plant	High	Medium
Leaf traits	Leaf area	•••	•••	••○	••○	•••	•○○	Organ/Plant	High	Medium
	Leaf angle	•••	••○	•○○	••○	••○	••○	Organ	High	Low
	Leaf length/width	•••	••○	•○○	••○	••○	•○○	Organ	High	Low
	Leaf thickness	•••	○○○	○○○	○○○	•○○	○○○	Organ	Medium	Not feasible
Canopy traits	Canopy height	••○	•••	••○	•••	•○○	•○○	Canopy	High	High
	Canopy volume	•○○	•••	••○	•••	•○○	•○○	Canopy	High	High
	Canopy cover	•○○	•••	••○	•••	•○○	•○○	Canopy/Pop	High	High
	LAI	••○	••○	••○	•••	•○○	•○○	Canopy	Medium	Medium
	Light interception	○○○	••○	•○○	•••	○○○	○○○	Canopy	Medium	Medium
Reproductive	Ear/panicle volume	•••	••○	•○○	••○	••○	•○○	Organ	High	Low
	Fruit count	••○	•••	••○	••○	•○○	•○○	Plant	Medium	Low
Stress response	Lodging assessment	○○○	•••	•○○	•••	○○○	○○○	Canopy/Pop	Low	High
	Wilting detection	••○	••○	•○○	••○	•○○	•○○	Plant	High	Medium
Root architecture traits										
Root traits	Root length	•••	••○	○○○	○○○	••○	○○○	Organ	High (rhizotron)	Not feasible*
	Root diameter	•••	•○○	○○○	○○○	••○	○○○	Organ	High (rhizotron)	Not feasible*
	Root angle	••○	••○	○○○	○○○	•○○	○○○	Organ	Medium	Not feasible*
	Root system architecture	••○	••○	○○○	○○○	•○○	○○○	Plant	High (CT/MRI)	Low (GPR/ERT)*

•••Highly suitable (validated accuracy, practical deployment); ••○Moderately suitable (achievable with limitations), •○○Limited suitability (significant constraints); ○○○Not suitable or not validated;

CCP/FCP ratings: High/Medium/Low/Not feasible

Field root phenotyping requires specialized technologies (GPR, ERT, excavation) not covered in this review’s scope.

Suitability ratings synthesize published validation studies and practical considerations. Technology abbreviations: LTS, Laser Triangulation; MVS, Multiview Stereo; ToF, Time-of-Flight; TLS, Terrestrial Laser Scanning; SL, Structured Light; LF, Light Field. Optimal scale indicates the spatial resolution at which each trait is typically assessed. CCP and FCP suitability reflect both achievable accuracy and practical deployment constraints in each environment.

#### 4.3.3 CCP versus FCP trait measurability

A critical consideration in phenotyping workflow design is that the range of measurable traits differs substantially between the CCP and FCP environments. Under controlled conditions, fine-scale organ traits (such as leaf thickness, leaf surface texture, and small reproductive structures) are accessible using high-precision sensing technologies. These measurements are often not achievable under field conditions because of the reduced resolution at greater sensing distances, environmental interference, and the inability to isolate individual organs within dense canopies.

Conversely, certain traits manifest differently or are only meaningful under specific field conditions. For example, lodging susceptibility requires wind and water stress in field environments. CCP assessments of lodging-related traits (stem diameter, plant height, and root anchorage) provide only indirect indicators of the actual lodging risk. Similarly, canopy-level light interception dynamics, competitive plant–plant interactions, and responses to natural stress gradients require FCP assessment for agronomically relevant characterization.

This complementarity reinforces the need for integrated phenotyping strategies that leverage CCP for high-precision organ-level characterization and algorithm development while using FCP to validate trait expression and assess genotype-by-environment interactions under realistic conditions (Poorter et al., 2016). The technology-trait mapping provided in this study can guide researchers in selecting appropriate sensing solutions for each component of the integrated workflows.

As the technology-trait mapping above illustrates, quantifying both canopy and root architecture remains a significant challenge in plant phenotyping, particularly under field conditions, owing to the inherent complexity and variability of plant structures. The suitability ratings in **Table 3** reflect not only the sensor capabilities but also the practical constraints that emerge when transitioning from controlled to field environments in real-world applications. With its intricate interplay of traits, canopy architecture presents difficulties in measurement because of environmental factors such as light variability and wind in field conditions, as well as plant density and dynamic spacing in the field. Similarly, root architecture is notoriously difficult to assess because of its hidden nature, soil heterogeneity, and the destructive nature of traditional excavation methods. While manual measurement remains common in both cases, it is labor-intensive, time-consuming, and lacks precision.

However, recent advancements in 3D phenotyping technologies have revolutionized the study of canopy and root structures offering non-invasive and scalable solutions that provide high-resolution data. In CCP environments, technologies such as LiDAR (Cao et al., 2017), photogrammetry (Gao et al., 2024; D. Yang et al., 2024), rhizotrons (Lobet et al., 2011), and X-ray computed tomography (Wu and Guo, 2014) facilitate detailed and accurate trait measurements. These environments allow for the isolation of individual plants and the creation of stable and consistent conditions, making it easier to achieve precision. For canopy architecture, this implies capturing detailed plant morphologies under consistent lighting. In contrast, technologies such as transparent soil systems and hydroponics enable the study of root development with minimal interference. However, despite their advantage CCP measurements may not fully replicate the complexities of natural environments, limiting the applicability of these findings to field conditions.

In contrast, FCP presents significant challenges for both canopy and root architecture measurements, but is crucial for understanding crop performance under real-world conditions. In field settings, environmental variability, soil heterogeneity, and plant interactions complicate the measurement consistency and accuracy of measurements. Canopy phenotyping in the field benefits from the use of UAVs, quadruped robotics, and LiDAR, enabling large-scale data collection across entire crop fields. These technologies offer practical solutions for medium- to high-resolution canopy measurements in dynamic outdoor settings. For root phenotyping, advanced tools such as ground-penetrating radar (GPR) (Liu et al., 2018; Lombardi et al., 2021) and electrical resistivity tomography (ERT) (Peruzzo et al., 2020) are helping researchers overcome the difficulties of non-invasive root system analysis. These field-deployed technologies enable large-scale, high-resolution phenotyping without disrupting the plant–soil system, offering more accurate reflections of natural root development.

In both canopy and root architecture studies, integrating advanced imaging technologies is critical for bridging the gap between precision in controlled environments and field scalability. Although field-based phenotyping poses greater technical challenges, it is indispensable for evaluating the actual performance of crop varieties under realistic growing conditions, and continued advancements in sensor technologies will further enhance our ability to capture these complex phenotypes.

## 5 Discussions and future perspectives

### 5.1 Overview of key findings

This review systematically compared 3D phenotyping technologies across Chamber-Crop Phenotyping (CCP) and Field-Crop Phenotyping (FCP) environments, revealing the fundamental trade-offs that shape technology selection for different research and breeding objectives. Additionally, this synthesis provides cross-cutting insights that can guide practitioners in selecting appropriate solutions for their specific phenotyping requirements.

The most consistent finding across all technologies was an inverse relationship between measurement precision and operational scalability. In CCP environments, technologies such as laser triangulation (LTS) and structured light (SL) achieve micrometer-level accuracy (14 µm–45 µm for LTS; Paulus et al., 2014), enabling detailed organ-level measurements, including petal thickness, leaf surface geometry, and fine-scale growth dynamics of plants. However, these high-precision approaches are fundamentally limited to single-plant or small-batch applications because of their short operational range, sensitivity to environmental conditions, and time-consuming nature of data acquisition protocols.

Conversely, FCP-oriented deployments sacrifice fine-scale precision for field-relevant scalability. UAV-mounted MVS and mobile TLS systems can cover hectares within hours, achieving centimeter-level accuracy sufficient for canopy-level trait extraction (R² = 0.78–0.99 for height and biomass estimates; Kim et al., 2021; Zhu et al., 2021). This precision degradation, from micrometers to centimeters, reflects not only sensor limitations but also the compounding effects of environmental variability, platform instability, and increased measurement distances inherent to field operations.

A critical insight from this comparative analysis is that environmental robustness, rather than theoretical accuracy, often determines the practical suitability of a technology. Technologies that exhibit high performance in controlled settings may fail catastrophically under field conditions. Structured light sensors, for example, achieve excellent results in laboratory environments (<13 mm error; Nguyen et al., 2015), but are severely compromised by ambient sunlight, restricting their field deployment to dawn, dusk, or nighttime operations (Rosell-Polo et al., 2015). Similarly, light-field cameras demonstrate interesting capabilities for post-capture refocusing but suffer from a limited effective depth range (10 cm–50 cm) and computational demands that preclude routine field applications (Schima et al., 2016).

In contrast, terrestrial laser scanning (TLS) and LiDAR-based approaches exhibit superior robustness across lighting conditions because of their active illumination and independence from ambient light. This environmental resilience, combined with mature processing algorithms and commercial availability, explains the growing adoption of TLS for field phenotyping, despite the higher equipment costs. The recent development of backpack-mounted LiDAR systems (Zhu et al., 2021) represents a significant advancement in bridging the gap between TLS precision and field-scale mobility.

Rather than viewing CCP and FCP as competing approaches, this review highlights their fundamentally complementary roles in phenotyping pipelines. CCP environments remain essential for (i) early stage trait discovery and method development, where environmental control enables the isolation of specific treatment effects, (ii) high-precision validation of genotype–phenotype associations requiring organ-level measurements, and (iii) algorithm training and sensor calibration prior to field deployment.

FCP provides irreplaceable value for (i) evaluating genotype-by-environment interactions under realistic growing conditions, (ii) capturing population-level variation across large breeding trials, and (iii) assessing traits that only manifest under field stresses, including wind, variable irrigation, and natural pest pressure. The poor correlation between controlled environment and field phenotypic data documented by Poorter et al. (2016), a meta-analysis finding that forms a sobering backdrop for this review, underscores that neither approach can substitute for the other.

The synthesis of the current literature reveals an emerging trend toward multi-sensor integration and data fusion approaches. Combining TLS or LiDAR structural data with hyperspectral or thermal imaging enables the simultaneous capture of geometric and physiological traits, providing a more comprehensive phenotypic characterization than any single modality (Dilmurat et al., 2022; Huang et al., 2018). This multi-sensor paradigm addresses a key limitation of geometry-only phenotyping: the inability to directly assess plant physiological status using structural data alone.

Based on this comparative analysis, we offer the following guidance for technology selection: (i) for organ-level trait measurement requiring micrometer precision, laser triangulation or high-end MVS systems in controlled environments remain optimal; (ii) for plot-level field phenotyping emphasizing throughput and environmental robustness, TLS (including mobile and backpack configurations) or UAV-based MVS provide the best balance of accuracy and scalability; (iii) for real-time monitoring applications requiring high temporal frequency, ToF cameras offer advantages in acquisition speed despite lower spatial resolution; and (iv) for cost-constrained applications, low-cost MVS systems using consumer-grade cameras provide accessible entry points, although with increased processing requirements and reduced accuracy compared to active sensing alternatives.

The quantitative synthesis presented in **Table 1** provides a structured reference for these comparisons, enabling researchers to identify technologies that match their specific requirements for accuracy, throughput, platform compatibility, and environmental conditions.

### 5.2 Temporal resolution and throughput considerations

Beyond spatial accuracy and trait coverage, temporal resolution, the frequency at which measurements can be repeated, is a critical but often underappreciated technological characteristic. The ability to capture plant growth dynamics, diurnal patterns, and stress responses fundamentally depends on the measurement frequency, which varies substantially across different technologies and platforms (**Table 4**).

**Table 4:** Temporal characteristics of 3D phenotyping technologies.

Technology	Single-plant acquisition	Plot-level acquisition	Field-scale throughput	Limiting factor
LTS	1 min–10 min	10 min–30 min	<1 ha/day	Scan time, repositioning
MVS	1 min–5 min (multi-view)	5 min–15 min	20 ha/day–100 ha/day (UAV)	Processing, flight time
ToF	Real-time	Real-time + movement	5 ha/day–20 ha/day (robot)	Platform speed
TLS	5 min–30 min	30 min–60 min (multi-position)	5 ha/day–30 ha/day (mobile)	Scan positions, data volume
SL	<5 s	1 min–5 min	N/A (CCP only practical)	Lighting conditions
LF	<1 s	1 min–5 min	N/A (experimental)	Processing, limited range

Acquisition times assume standard sensor configurations and exclude data processing time. Field-scale throughput assumes appropriate platform (UAV for MVS, mobile platform for TLS). Real-time acquisition enables video-rate capture but generates substantial data volumes requiring downstream processing. Actual throughput varies with experimental design, crop characteristics, and operator experience.

At one extreme, time-of-flight cameras and RGB video systems enable continuous real-time acquisition at 30–60 frames per second, supporting the analysis of rapid plant movements, including leaf heliotropism, nyctinasty, and wind-induced motion (Biskup et al., 2007). Such a high temporal resolution is achievable only for stationary single-plant setups in CCP environments. At the other extreme, UAV-based photogrammetric surveys of large field trials may be conducted weekly or bi-weekly, constrained by flight planning, weather windows, and data processing capacity, rather than by fundamental sensor limitations.

Between these extremes, most phenotyping systems operate at intermediate temporal resolutions determined by the acquisition time, repositioning requirements, and processing throughput. Fixed gantry systems, such as the Field Scanalyzer, can achieve daily or twice-daily scans of experimental plots (Virlet et al., 2016), enabling the detection of growth rate differences and stress onset. Mobile ground platforms typically achieve plot revisit intervals of 2–7 days for large breeding trials. The critical trade-off involves spatial resolution versus temporal frequency: systems optimized for detailed organ-level measurements generally sacrifice throughput, whereas high-throughput field systems sacrifice spatial detail.

For dynamic phenotyping applications, such as tracking growth rates, stress responses, or developmental transitions, temporal resolution may outweigh spatial resolution in importance. A 10-day measurement interval may entirely miss critical growth windows, whereas daily measurements at reduced spatial resolution can capture phenological differences essential for breeding selection. This trade-off should explicitly inform technology and platform selection based on the specific biological questions being addressed, with growth-rate-sensitive applications prioritizing temporal frequency and morphological characterization prioritizing spatial details.

### 5.3 Technological advancements

Over time, technological advancements have significantly enhanced the capabilities of 3D phenotyping in both CCP and FCP environments. In particular, the development of high-resolution 3D sensing technologies, such as LiDAR and SL systems, has enabled precise and comprehensive data collection across various plant traits. These sensors enable the generation of detailed 3D models of crops, capturing key features such as plant height, canopy volume, and biomass distribution, which are critical for evaluating crop performance and health.

In CCP, the integration of laser triangulation and SL systems has been particularly effective for close-range phenotyping (Lee et al., 2013; Nguyen et al., 2016b). These systems excel in controlled environments, offering high accuracy in capturing minute morphological changes. The articulated arm and fixed-post mounts commonly used in CCP setups further enhance precision by allowing the sensors to maintain consistent positioning and scanning parameters, enabling repeatable and reliable measurements.

Advancements in sensor carrier platforms, such as drones and wheeled robots, have revolutionized data collection in the field. Drone-mounted LiDAR and photogrammetry systems have enabled rapid large-scale data acquisition, providing high-resolution 3D maps of entire fields within minutes (Gano et al., 2024). Similarly, wheeled and treaded robots equipped with terrestrial laser scanners offer detailed ground-level 3D imaging, making it possible to capture lower canopy structures and root zone traits that are often missed by aerial platforms (Iqbal et al., 2020b). These platforms improve spatial coverage and allow real-time data collection, offering insights into crop growth dynamics.

One critical advancement that has emerged is the fusion of multi-sensor system data, where 3D imaging technologies are combined with other data modalities, such as hyperspectral or thermal imaging (Dilmurat et al., 2022; Huang et al., 2018). This data fusion enables a more holistic view of crop performance, allowing researchers to correlate 3D structural data with physiological traits, such as leaf chlorophyll content or water stress, leading to more accurate phenotyping. Additionally, improved algorithms for data processing, particularly in handling large datasets from field deployments, are helping to overcome issues of noise and occlusion, resulting in cleaner and more interpretable data (Kahraman and Bacher, 2021).

The adoption of AI-driven image analysis tools and machine learning algorithms has further refined the ability to extract meaningful insights from 3D phenotypic data sets. These technologies automate the classification of complex traits, reduce human error, and enable high-throughput phenotyping at previously unattainable scales (Feng et al., 2024; Lu et al., 2023; Zhang et al., 2021). As these tools continue to develop, their integration into both CCP and FCP systems will be critical for accelerating the speed and accuracy of crop trait evaluation.

### 5.4 Error sources and mitigation strategies

Understanding the sources of measurement errors in 3D phenotyping is essential for appropriate technology selection, experimental design, and result interpretation. Although error sources have been discussed throughout the technology-specific sections of this review, a unified taxonomy provides a framework for systematic comparisons and targeted mitigation. Following the categorization approach of Harandi et al. (2023) and Paulus (2019), we classified the error sources into three primary categories: sensor-intrinsic, scene-related, and environment-related errors.

#### 5.4.1 Sensor-intrinsic errors

Sensor-intrinsic errors arise from the fundamental limitations of sensing hardware and associated signal processing. These include spatial resolution limits, as each technology has characteristic resolution constraints, such as micrometer-level for laser triangulation, millimeter-level for structured light and high-end MVS, and centimeter-level for ToF and field-deployed TLS. These limits define the minimum feature size that can be reliably detected and directly constrain the traits accessible to each of the technologies. Depth accuracy and noise: Depth measurements are subject to systematic biases and random noise that vary with distance, surface orientation, and the material properties. ToF cameras exhibit characteristic “flying pixel” artifacts at depth discontinuities (Kazmi et al., 2014), whereas laser triangulation systems exhibit depth-dependent accuracy variations within their operational range (Paulus et al., 2014). Calibration errors: Multi-camera MVS systems and structured light projector-camera pairs require precise geometric calibration; residual calibration errors propagate through the reconstruction pipeline, causing systematic distortions that may exceed sensor noise in poorly calibrated systems.

#### 5.4.2 Scene-related errors

Scene-related errors arise from interactions between the sensing modality and the characteristics of plant targets: Occlusion and incompleteness: Plant self-occlusion is ubiquitous, with leaves, stems, and reproductive structures blocking sensor views to underlying structures. This affects all technologies but is particularly problematic for single-viewpoint sensing applications. The severity of this phenomenon depends on the plant architecture, growth stage, and viewing geometry. Surface optical properties: Leaf reflectance varies with chlorophyll content, surface texture, and moisture status, affecting laser triangulation accuracy (Dupuis et al., 2015) and MVS feature-matching success. Specular (shiny) and translucent surfaces present particular challenges, potentially causing systematic depth errors or reconstruction failures. Texture and feature availability: Passive MVS techniques require detectable surface features for correspondence matching to be effective. Uniform, texture-less surfaces, which are common on young leaves, stems, and some fruits, can cause sparse or failed reconstruction in the affected regions. Plant motion during acquisition: Non-rigid plant motion during scanning violates the static scene assumptions underlying most reconstruction algorithms. Even subtle motion between frames can cause blurring, misalignment, or spurious geometries in the resulting point cloud.

#### 5.4.3 Environment-related errors

Environment-related errors reflect the influence of external conditions on sensing performance. Ambient lighting: Structured light and ToF cameras are highly sensitive to ambient illumination, with direct sunlight capable of completely overwhelming projected patterns or modulated signals. Even passive MVS experiences reduced accuracy under variable lighting owing to feature matching inconsistencies (Paturkar et al., 2019). Wind effects: Wind-induced plant motion represents a primary environmental constraint for field phenotyping, affecting all technologies that require temporal integration (scanning systems) or multi-image acquisition (MVS). The severity is scaled according to wind speed, plant flexibility, and acquisition duration. Atmospheric conditions: Dust, fog, rain, and humidity affect laser propagation and camera optics, introducing noise or complete sensing failure under adverse conditions. These effects are generally more severe for active sensing modalities that rely on laser return signals.

**Table 5** provides a systematic mapping of error source severity across the six technologies reviewed, synthesizing the technology-specific discussions in *Section 2*. This framework can guide technology selection based on the anticipated operating conditions and inform the design of mitigation strategies, such as multi-viewpoint acquisition, environmental controls, or robust reconstruction algorithms.

**Table 5:** Error source severity by technology and environment.

Error category	Error source	LTS	MVS	ToF	TLS	SL	LF	CCP impact	FCP impact
Sensor-intrinsic	Spatial resolution limits	Low	Med	High	Med	Med	High	Med	High
	Depth noise/accuracy	Low	Med	Med	Low	Med	High	Low	Med
	Calibration sensitivity	Med	High	Low	Low	High	High	Med	Med
Scene-related	Occlusion/incompleteness	High	High	High	Med	High	High	Med	High
	Surface reflectance effects	High	Med	Med	Med	Med	Low	Med	Med
	Texture-less surfaces	N/A	High	N/A	N/A	N/A	Med	Med	Med
	Plant motion blur	Med	High	Low	Med	Med	Low	Low	High
Environment-related	Ambient light interference	Low	Med	High	Low	High	Low	Low	High
	Wind-induced motion	Med	High	Med	Med	Med	Med	Low	High
	Atmospheric conditions	Low	Med	Med	Med	Med	Low	Low	Med

High, Major limitation, significant accuracy degradation

Med, Moderate impact, mitigation typically required

Low, Minor impact, generally manageable

N/A, Not applicable to this technology

CCP/FCP, Impact indicates typical severity in each environment

Severity ratings synthesize published validation studies and reflect typical operating conditions. Actual impact varies with specific sensor models, plant species, and experimental protocols. Technologies with active illumination (LTS, TLS) are generally less affected by ambient light but more affected by surface optical properties.

### 5.5 Technology integration

Rapid advancements in imaging technologies, such as SL, LF cameras, TLS, ToF cameras, and multiview stereo, have individually contributed significantly to plant phenotyping. Each of these technologies, as discussed in the previous sections, offers unique capabilities that address different aspects of plant analysis, from capturing detailed 3D structures to measuring precise distances and reflecting plant morphology in diverse environments. However, the true potential of these technologies can be realized when they are combined, creating a synergistic approach that enhances the accuracy, resolution, and efficiency of phenotyping.

The integration of these phenotyping technologies can allow researchers to leverage the strengths of each approach and compensate for the limitations of the others. For instance, although ToF cameras provide accurate distance measurements and are effective in dynamic environments (Kazmi et al., 2014; Song et al., 2011), they might lack the fine details captured by LF Cameras (Polder and Hofstee, 2014). Conversely, LF Cameras excel in capturing intricate details and enabling post-capture refocusing, but they may not perform as well in large-scale field applications, where TLS provides broader coverage and robust 3D mapping (Schima et al., 2016).

By combining these technologies, researchers can establish a comprehensive phenotyping pipeline. For example, TLS can create large-scale, high-resolution 3D models of entire plant canopies or fields, capturing structural data at the macro level. This data can then be complemented by the fine-scale detail obtained from LF Cameras, which can focus on individual plants or specific traits within the canopy. Additionally, the integration of ToF cameras allows for real-time data collection in dynamic environments, making it possible to monitor changes in plant phenotypes as they occur.

The combined use of these imaging technologies opens new possibilities for controlled indoor phenotyping and large-scale field studies. In controlled environments, such as greenhouses or growth chambers, the integration of SL with Multiview Stereo systems can facilitate a detailed analysis of plant structures, including leaf morphology, stem thickness, and flower development (Nguyen et al., 2016a). The combination of TLS and ToF depth cameras is particularly powerful in field applications. TLS can provide detailed 3D models of plant populations across entire fields, whereas ToF depth cameras can capture dynamic changes in plant growth and responses to environmental conditions over time (Shafiekhani et al., 2017). This integration allows for the monitoring of large-scale phenotypic traits, such as canopy height, biomass distribution, and spatial variability, within a crop field. Moreover, these combined datasets can be fed into machine learning models to predict yield, assess stress responses and guide precision agriculture practices (Shafiekhani et al., 2017).

Although integrating these advanced imaging technologies offers significant benefits, it also presents challenges. One of the main challenges is the need for sophisticated data fusion techniques that combine datasets from different modalities into coherent and interpretable models. Differences in spatial resolution, data formats, and temporal scales must be reconciled to ensure an accurate and meaningful analysis. Additionally, the high volume of data generated by these combined technologies requires efficient processing and storage solutions, as well as robust algorithms for extracting relevant phenotypic information.

### 5.6 Remaining challenges: the scalability-accuracy trade-off and phenotyping prioritization

#### 5.6.1 The central trade-off

The analysis presented in this review converges on a fundamental tension that pervades 3D plant phenotyping: the inverse relationship between measurement precision and operational scalability. This scalability-accuracy trade-off is not merely a technical limitation but represents a core design constraint that shapes technology selection, experimental design, and the scope of phenotypic questions that can be addressed practically.

**Figure 9:**
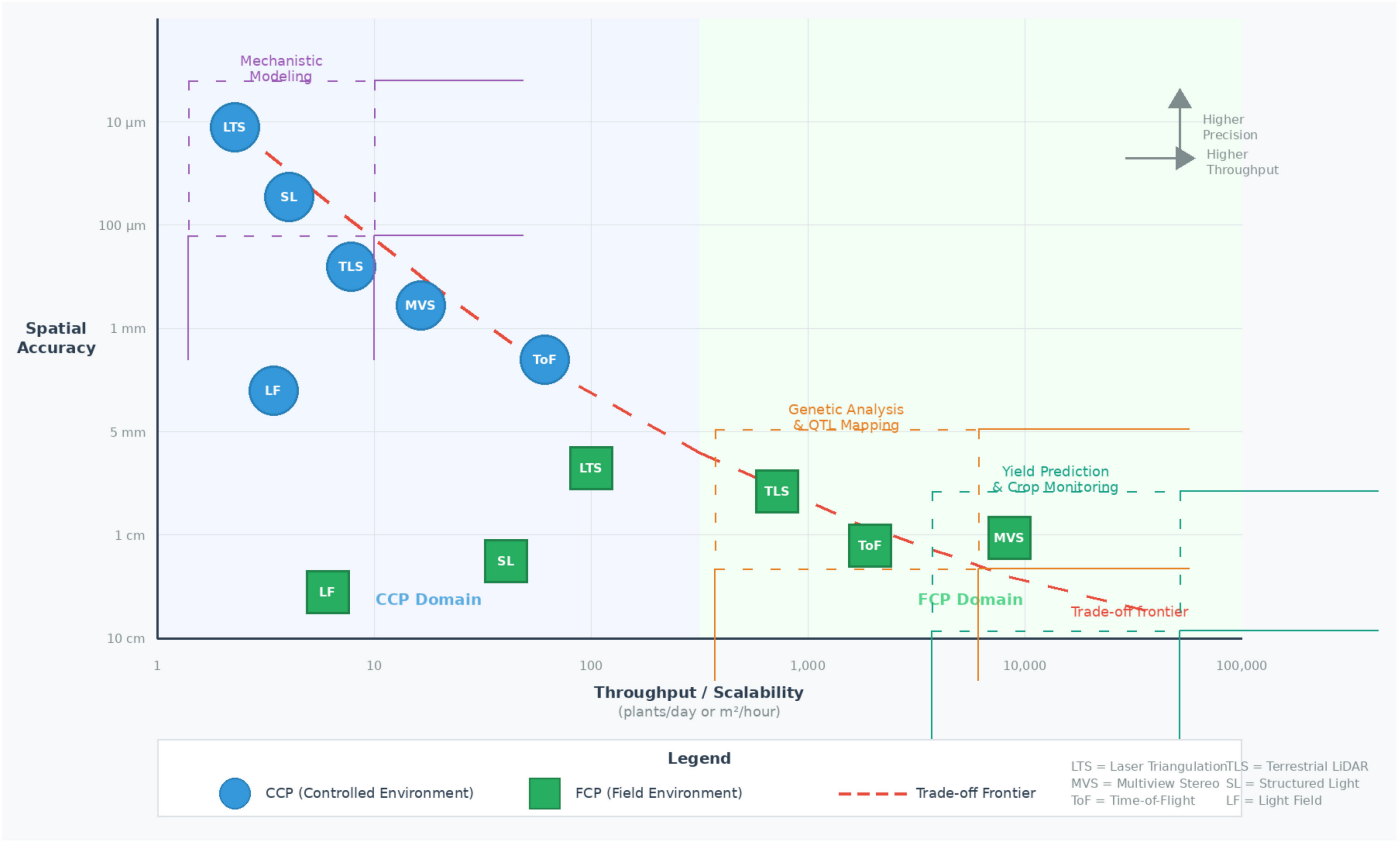
Scalability-accuracy trade-off in 3D plant phenotyping, technology-platform combination across CCP and FCP environments.

At one extreme, laboratory-based laser triangulation systems achieve micrometer-level precision suitable for detecting subtle morphological differences; however, their throughput is limited to individual plants scanned over several minutes. At the other extreme, UAV-based photogrammetry can survey hundreds of hectares daily, but the achievable precision degrades to centimeters, which is adequate for canopy-level traits but insufficient for organ-level trait characterization. Between these extremes lies a continuum of technology-platform combinations, each representing a specific position on the scalability-accuracy curve (**Figure 9**). The critical insight is that no single system can simultaneously maximize both dimensions; rather, practitioners must select technologies that match their specific precision requirements and throughput constraints.

#### 5.6.2 Objective-based technology selection framework

To operationalize this trade-off, we propose a decision framework that maps common phenotyping objectives to the recommended technology and platform combinations (**Table 6**). This framework recognizes that different research questions and stakeholder needs require different positions on the scalability-accuracy continuum.

**Table 6:** Objective-based technology selection framework for 3D phenotyping.

Objective	Priority traits	Precision required	Throughput required	Recommended technology	Recommended platform	Environment (CCP/FCP)
Genetic mapping/QTL analysis	Height, biomass, flowering time, canopy cover	cm-level	High (>500 plots)	MVS, TLS	UAV, mobile ground	FCP
Yield prediction	Height, LAI, canopy volume, NDVI	cm-level	High (field-scale)	MVS, TLS	UAV	FCP
Growth rate monitoring	Height change, leaf area expansion	cm-level	Medium-High (daily)	TLS, MVS	Ground robot, Fixed gantry, UAV	CCP, FCP
Stress detection (acute)	Wilting, leaf angle, canopy temperature	cm-level	High (hourly-daily)	ToF, MVS, TLS	Fixed gantry, ground robot	CCP preferred
Stress detection (chronic)	Height reduction, biomass, senescence	cm-level	Medium	MVS, TLS	UAV, mobile ground	FCP
Organ-level morphology	Leaf dimensions, stem diameter, internode length	mm-level	Low (<100 plants)	LTS, SL, MVS	Articulated arm, turntable	CCP
Mechanistic modeling	Detailed architecture, organ geometry	µm–mm level	Very Low	LTS, MVS (multi-view)	Turntable, articulated arm	CCP
Lodging assessment	Canopy angle, height heterogeneity	cm-level	High	MVS, TLS	UAV	FCP
Root phenotyping	Root architecture, depth distribution	mm-level	Low	CT, MRI (CCP); GPR (FCP)	Specialized systems	CCP, FCP
Commercial crop monitoring	Canopy health, uniformity, biomass	cm-level	Very High	MVS	UAV (consumer)	FCP

Precision categories: µm, micrometer (organ detail); mm, millimeter (organ-level); cm, centimeter (plant/canopy level). Throughput categories: Very Low (<50 plants/day), Low (<100), Medium (100–500), High (>500 plots/day), Very High (commercial field scale). Recommendations represent general guidance; optimal selection depends on specific experimental design, available resources, and local conditions.

For genetic analysis and QTL mapping, where the goal is to detect phenotypic differences between genotypes, moderate precision is typically sufficient, as genetic effects manifest as population-level differences rather than individual-plant variation. UAV-based MVS or mobile TLS platforms provide adequate accuracy for most canopy-level traits while enabling the throughput necessary for statistically powered genetic studies (hundreds to thousands of plots).

For yield prediction and crop modeling, the emphasis shifts toward canopy-level traits (height, LAI, and biomass) that correlate with the final yield. UAV platforms excel in this regard, with daily or weekly acquisitions enabling time-series analyses that capture growth dynamics. The reduced precision of aerial systems is acceptable because yield prediction models typically operate at the plot or field scale.

For stress detection and response characterization, temporal resolution often outweighs spatial resolution. The ability to capture rapid physiological responses (e.g., wilting, leaf angle changes, and growth rate alterations) requires measurement frequencies that may only be achievable with fixed gantry systems or continuous-monitoring robotic platforms. Waiting for weekly UAV surveys may result in the omission of critical stress events.

For mechanistic modeling and physiological research, organ-level measurements (leaf dimensions, stem architecture, and reproductive structure counts) require high-precision, which is achievable only in controlled environments. These applications accept low throughput as a necessary trade-off for the detailed measurements required to parameterize and validate physiological models.

#### 5.6.3 Trait prioritization in field conditions

Beyond technology selection, the scalability-accuracy trade-off raises fundamental questions about which traits should be measured under field conditions. Not all traits measurable in controlled environments can or should be measured on the field scale. The decision of which traits to prioritize involves balancing the measurement feasibility with biological and practical relevance.

For crops with dense canopies (soybean, rice, and maize at late growth stages), individual plant measurements may be both technically challenging due to occlusion and practically unnecessary if plot-level traits capture the relevant variation. Canopy-level measurements—height, cover, LAI, and biomass indices—may provide equivalent predictive power for breeding selection while requiring dramatically less measurement effort.

Conversely, some traits that are difficult to measure directly can be inferred from more accessible measurements. Biomass, for example, can be estimated from height and canopy volume with sufficient accuracy for many applications, avoiding the need for destructive sampling. Similarly, stress responses may be detectable through canopy structural changes before they manifest as yield differences.

#### 5.6.4 Stakeholder-specific considerations

Different stakeholders operate at different positions on the scalability-accuracy continuum based on their specific needs. For production-scale farmers, detailed organ-level measurements across entire fields are neither feasible nor necessary, and actionable information regarding crop health, growth uniformity, and stress occurrence can be derived from canopy-level observations. Sampling strategies and detailed measurements of representative plots or plants while surveying the broader field at a lower resolution may provide the most practical approach.

For plant breeders, priorities vary according to the experimental stage. Early generation selection in controlled environments may require detailed measurements to identify subtle trait differences, whereas advanced yield trials under field conditions may prioritize throughput to evaluate large numbers of lines across multiple environments. Understanding these stage-specific requirements is essential for designing phenotyping workflows that meet the needs of breeding program.

#### 5.6.5 Bridging technology and application

A persistent gap exists between technological development and agricultural applications. Engineers and data scientists developing phenotyping systems may not fully understand which traits are most relevant for breeding or agronomy, whereas breeders and agronomists may not appreciate the technical constraints that determine what is measurable at different scales. Addressing this gap requires sustained interdisciplinary collaboration to ensure that phenotyping tools are aligned with actual user needs, rather than technical capabilities alone.

#### 5.6.6 Remaining technical challenges

In addition to the scalability-accuracy trade-off, significant technical challenges persist. Environmental factors, such as wind, rain, dust, and variable lighting, continue to degrade data quality under field conditions. Platform stability on uneven terrain affects sensor precision. The computational demands for processing terabytes of 3D data strain the available infrastructure. Although sensor costs are decreasing, they remain prohibitive for many potential users. Data interoperability between different sensor systems and analysis pipelines limits integration. The challenge of scaling from plot-level experiments to farm-level implementation remains largely unresolved. These challenges represent active areas of research and development, with advances in each area incrementally expanding the practical envelope of field-based, 3D phenotyping.

### 5.7 Future perspectives in 3D field crop phenotyping

The future of 3D crop phenotyping is rapidly evolving, with emerging technologies that promise to address existing challenges and expand the potential of phenotyping systems. Soft robots and sensors represent a breakthrough in flexibility, adaptability, and safety in handling delicate plants. Unlike rigid robots, soft robots constructed from flexible materials can move fluidly through dense crops and gently interact with plants without causing damage (Del Dottore et al., 2024). For example, soft robotic arms can be equipped with soft sensors to measure traits such as leaf thickness, stem strength, and fruit ripeness, providing valuable phenotypic data without harming plants. These systems are particularly well-suited for environments where traditional robotic systems might struggle, such as in tight or uneven planting arrangements.

Simultaneously, the development of quadruped robots is transforming data collection in challenging field environments. Unlike wheeled or treaded robots, quadrupeds can navigate rugged terrains, such as hilly fields or areas with dense vegetation, where other robotic platforms experience mobility constraints. These four-legged robots offer stability, precision, and flexibility, allowing them to carry sensors into areas that are otherwise difficult to access (Lopes et al., 2023). Equipped with 3D imaging systems, quadruped robots can collect detailed data on plant architecture, leaf orientation, and canopy structures across varying terrains. This technology opens the door to more comprehensive data collection in real-world agricultural settings, particularly in locations where traditional wheeled robots cannot operate efficiently.

An exciting frontier is the synchronization of drones and ground robots for more coordinated and efficient phenotyping. Drone-ground robot synchronization allows for real-time collaboration between aerial and ground-based sensor platforms, combining the strengths of both systems (Chai et al., 2024; Güler and Yıldırım, 2023). For example, drones could provide a high-level overview of the field, capturing large-scale 3D data on canopy structure and spatial variability, while ground robots can perform close-up measurements on individual plants, focusing on more detailed traits such as stem diameter, fruit size, or root exposure. By working in sync, these systems can collect multi-scale phenotypic data more efficiently, covering larger areas while maintaining the precision required for detailed trait analysis. The integration of real-time feedback loops between drones and ground robots also enhances the ability to optimize data collection strategies, dynamically adjusting sensor positioning or targeting specific areas of interest (Chai et al., 2024).

The emergence of digital twins for automated real-time 3D field plant phenotyping has added to this technological horizon. Digital twins, which are virtual replicas of physical systems, enable the real-time modeling and analysis of plant growth and behavior under various scenarios. Leveraging advanced 3D functional plant modeling frameworks (Mitsanis et al., 2024), these systems integrate phenotypic data, environmental conditions, and predictive models to dynamically simulate and monitor plant development. The applications of digital twins extend to understanding genotype-by-environment interactions, stress response prediction, and optimizing crop management strategies. Recent studies, such as those by Liu et al. (2024), have demonstrated how functional-structural plant modeling can form the basis of digital twins by combining phenotypic traits and environmental data to enhance decision-making in crop breeding and precision agriculture. By offering a platform for continuous monitoring and virtual experimentation, digital twins are poised to bridge the gap between research and practical applications, making them a transformative tool in field-based phenotyping.

Another critical advancement is 3D spectral fusion, which goes beyond traditional geometry-based phenotyping by combining 3D structural data with spectral information obtained from hyperspectral and multispectral imaging. This fusion of data modalities allows researchers to capture both the morphological characteristics of plants (e.g., plant height and canopy shape) and their physiological status (e.g., nutrient levels, chlorophyll content and water stress) (Dilmurat et al., 2022). By integrating geometry with spectral data, 3D spectral fusion provides a more holistic understanding of plant health and performance, enabling the identification of subtle stress indicators that may not be detectable using geometry alone. This approach has the potential to revolutionize the monitoring and management of crops, offering precise multidimensional insights into plant responses to environmental factors, diseases, and nutrient availability.

In addition to these technological advancements, edge computing is expected to play a transformative role in real-time data processing for field phenotyping. As the volume of data generated by 3D imaging systems grows exponentially, particularly in large-scale field applications, the ability to process data at the source rather than transferring raw data to the cloud will be critical. Edge computing allows localized data processing near the point of collection, enabling real-time analysis and reducing the overhead associated with transferring large datasets to the cloud for storage and analysis (Syu et al., 2023). This approach minimizes bandwidth usage, accelerates decision-making, and ensures that only relevant filtered data are sent to cloud systems for further processing.

The emergence of high-performance miniaturized hardware, such as edge computing devices produced by commercial entities (Scalcon et al., 2024), has made it feasible to perform complex computations at the edge. These low-power devices, equipped with AI-powered processors and GPU acceleration, can run machine learning models directly on drones, robots, or field stations, enabling real-time image analysis, trait detection, and anomaly identification (Scalcon et al., 2024). By processing data on-site, these systems can generate immediate insights into plant health, growth, and performance, which is especially useful for farmers or breeders who need to make quick, informed decisions about interventions such as watering, fertilization, or pest control.

Edge computing also addresses the scalability challenges associated with processing terabytes of 3D data across large areas. In situations where cloud connectivity may be limited, such as in remote or rural farming locations, edge devices can function independently, ensuring that data collection and processing continue without interruption. Additionally, edge computing offers enhanced data security because sensitive crop data can be processed and stored locally, reducing the risk of data breaches associated with cloud-based systems (Syu et al., 2023).

The combination of cloud platforms and edge computing provides a balanced solution for large-scale phenotyping. While cloud computing is essential for long-term storage, cross-field comparisons, and advanced analytics, edge computing optimizes on-the-fly processing and enables real-time action in the field. This hybrid approach ensures that phenotyping systems are efficient and practical for large-scale agricultural operations. Moreover, with emerging communication and processing architectures, such as edge learning for B5G (also known as 5G) networks with distributed signal processing demonstrated by Xu et al. (2023), semantic communication, edge computing, and wireless sensing are now possible over geographically dispersed edge nodes while minimizing the need for frequent data exchange.

In addition to new technologies, collaborative efforts between engineers, plant breeders, and agronomists will be crucial for refining and implementing 3D phenotyping systems. Engineers and computer scientists will need to work closely with breeders and agronomists to ensure that the technologies developed are relevant to the needs of real-world crop management and breeding programs. Interdisciplinary research will also help address the knowledge gap between technology developers and end users, ensuring that innovations in phenotyping technology are grounded in practical, field-relevant applications. This collaboration can guide the prioritization of critical traits that should be measured in different crops and environments, ensuring that the developed systems are both effective and efficient.

To realize the full potential of these innovations, scalability and standardization are essential. As technologies such as soft robotics, drone-ground robot synchronization, 3D spectral fusion, and edge computing continue to evolve, they must be adapted for large-scale applications in commercial farming. This requires advancements in sensor miniaturization, power efficiency, and real-time data processing, allowing these systems to be deployed over vast areas without compromising data quality. Additionally, the development of standardized protocols for sensor calibration, data collection, and analysis is necessary to ensure the reproducibility and interoperability of phenotyping tools across different research groups and agricultural systems.

## 6 Concluding remarks

This review explores significant advancements in 3D crop phenotyping technologies, emphasizing their roles in chamber crop phenotyping (CCP) and field crop phenotyping (FCP). While CCP offers precision and control in data collection, FCP provides the advantage of real-world applicability, addressing the complex environmental variability that crops face in actual agricultural settings. Together, these approaches form a complementary framework essential for high-throughput phenotyping and the development of resilient and high-performing crop varieties.

The introduction of advanced 3D sensing systems, such as TLS, LTS, SL and ToF cameras, has greatly enhanced our ability to capture detailed morphological and physiological traits. Furthermore, the integration of multi-sensor platforms and spectral fusion techniques has and is expected to further allow researchers to go beyond simple geometric measurements, offering a deeper understanding of plant health and performance than previously possible. When combined with AI-driven tools and machine learning algorithms, these technologies are pushing the boundaries of what can be achieved in automated phenotyping.

Despite these advancements, there are still significant challenges. Field phenotyping continues to grapple with environmental interference, sensor stability, and the sheer volume of data generated in large-scale applications. Addressing these challenges will require a combination of edge computing for localized, real-time data processing and cloud platforms for large-scale data storage and analysis. The emergence of high-performance miniaturized hardware by commercial entities will play a crucial role in ensuring that data processing becomes more efficient, scalable, and feasible for real-world applications in the future.

Looking forward, the continued development of autonomous robotic systems, soft sensors, and drone-ground synchronization will further enhance the precision, flexibility, and scalability of 3D phenotyping. As these technologies evolve, close collaboration between engineers, breeders, and agronomists will be vital to ensure that phenotyping tools are tailored to the practical needs of crop breeding and management programs in the future.

Ultimately, the future of 3D phenotyping lies in the ability to merge advanced technology with field applicability, enabling scalable, precise, and actionable insights into modern agriculture. As we overcome the current limitations and harness the potential of emerging innovations, 3D phenotyping will become a cornerstone of precision agriculture, driving sustainable improvements in crop yield, resilience, and food security.
